# Reconstructing the impact of COVID-19 on the immunity gap and transmission of respiratory syncytial virus in Lombardy, Italy

**DOI:** 10.1016/j.ebiom.2023.104745

**Published:** 2023-08-09

**Authors:** Hadrian Jules Ang, Francesco Menegale, Giuseppe Preziosi, Elena Pariani, Maurizio Migliari, Laura Pellegrinelli, Giuseppe Maria Sechi, Sabrina Buoro, Stefano Merler, Danilo Cereda, Marcello Tirani, Piero Poletti, Ilaria Dorigatti

**Affiliations:** a Medical Research Council Centre for Global Infectious Disease Analysis, School of Public Health, Imperial College London, London, United Kingdom; b Center for Health Emergencies, Fondazione Bruno Kessler, Trento, Italy; c Department of Mathematics, University of Trento, Trento, Italy; d Regional Agency for Innovation and Procurement, Milano, Italy; e Department of Biomedical Sciences for Health, University of Milan, Milan, Italy; f Agenzia Regionale Emergenza Urgenza, Milan, Italy; g Lombardy Region Welfare General Directorate, Milano, Italy; h Health Protection Agency of the Metropolitan Area of Milan, Milano, Italy

**Keywords:** RSV, Mathematical modelling, Catalytic models, Immunity gap, COVID-19 restrictions

## Abstract

**Background:**

Respiratory syncytial virus (RSV) is a leading cause of hospitalisation and mortality in young children globally. The social distancing measures implemented against COVID-19 in Lombardy (Italy) disrupted the typically seasonal RSV circulation during 2019–2021 and caused substantially more hospitalisations during 2021–2022. The primary aim of this study is to quantify the immunity gap-defined as the increased proportion of the population naïve to RSV infection following the relaxation of COVID-19 restrictions in Lombardy, which has been hypothesised to be a potential cause of the increased RSV burden in 2021–2022.

**Methods:**

We developed a catalytic model to reconstruct changes in the age-dependent susceptibility profile of the Lombardy population throughout the COVID-19 pandemic. The model is calibrated to routinely collected hospitalisation, syndromic, and virological surveillance data and tested for alternative assumptions on age-dependencies in the risk of RSV infection throughout the pandemic.

**Findings:**

We estimate that the proportion of the Lombardy population naïve to RSV infection increased by 60.8% (95% CrI: 55.2–65.4%) during the COVID-19 pandemic: from 1.4% (95% CrI: 1.3–1.6%) in 2018–2019 to 2.3% (95% CrI: 2.2–2.5%) before the 2021–2022 season, corresponding to an immunity gap of 0.87% (95% CrI: 0.87–0.88%). We found evidence of heterogeneity in RSV transmission by age, suggesting that the COVID-19 restrictions had variable impact on the contact patterns and risk of RSV infection across ages.

**Interpretation:**

We estimate a substantial increase in the population-level susceptibility to RSV in Lombardy during 2019–2021, which contributed to an increase in primary RSV infections in 2021–2022.

**Funding:**

10.13039/501100000265UK Medical Research Council (MRC), UK Foreign, Commonwealth & Development Office (FCDO), EDCTP2 programme, 10.13039/501100000780European Union, 10.13039/100010269Wellcome Trust, 10.13039/501100000288Royal Society, EU-MUR PNRR INF-ACT.

**Research in context**

**Evidence before this study**

We searched Medline using Ovid for all studies published up to 2 February 2023 with the query “(respiratory syncytial virus∗ OR RSV∗) AND (COVID∗ OR SARS-CoV-2∗)” and included the relevant subject headers “Respiratory Syncytial Viruses” and “COVID-19”. We found six studies employing dynamical or regression models projecting forward the expected impact of the easing of COVID-19 related non-pharmaceutical interventions (NPIs) on RSV cases or hospitalisations in the USA, South Africa, UK, Norway, and Japan. Changes in RSV circulation caused by the NPIs implemented to counter SARS-CoV-2 spread is hypothesised to be a key driver of observed changes in RSV burden.

**Added value of this study**

We analysed RSV surveillance data collated in Lombardy (Italy) throughout the COVID-19 pandemic, which adds to the growing list of geographical regions that have experienced an atypical RSV resurgence following the relaxation of COVID-19 restrictions. In this work we quantify the increase in population-level susceptibility to primary RSV infection from age-stratified disease surveillance and hospitalisation data. We also estimate age-dependencies in the risk of RSV infection, highlighting the variable impact that NPIs implemented against COVID-19 had on RSV transmission.

**Implications of all the available evidence**

Our findings contribute to better understanding age-dependencies in RSV transmission and susceptibility, and the effects that COVID-19 restrictions have had on other respiratory viruses such as RSV. The developed approach could be applied to different streams of surveillance data to estimate changes in the susceptibility profile of the population to other infectious diseases, which has implications for public health planning.

## Introduction

Respiratory syncytial virus (RSV) is a leading cause of acute lower respiratory infection (ALRI) in young children, which in severe cases can lead to hospitalisation and death.^1^, ^2^, ^3^ In 2019 alone, there were around 33 million RSV-associated ALRI episodes worldwide leading to around 100,000 deaths among children 0–5 years of age.^1^ Older adults are also vulnerable to severe disease due to RSV, which caused around 336,000 hospitalisations and 14,000 in-hospital deaths globally in 2015.^4^

RSV is transmitted through direct or indirect contact with secretions from the respiratory tract of an infectious individual, such as through large droplets or contaminated objects.^2^^,^^5^^,^^6^ Most children in their first months of life are protected against RSV infection by the passive transfer of maternal-specific antibodies, but titre levels vary across individuals.^2^^,^^5^ As antibody levels wane, infants become vulnerable to disease even if antibodies are still detectable.^2^^,^^5^^,^^7^

Infection provides short-term, partial immunity against reinfection,^8^ and long-term partial reduction in susceptibility to further post-primary infections.^9^^,^^10^ While symptoms among healthy adults are usually mild, in young children and vulnerable populations (e.g., those who are immunocompromised, and individuals with other pre-existing cardiovascular or pulmonary conditions) infection can develop into bronchiolitis or pneumonia and result in severe disease outcomes.^5^

While in tropical regions RSV seasonality is less well-defined,^10^ in temperate regions, such as Italy, RSV transmission is seasonal, starting in autumn and ending in spring.^11^^,^^12^ The emergence of the coronavirus disease 2019 (COVID-19) pandemic prompted the introduction of non-pharmaceutical interventions (NPIs).^13^ A retrospective analysis of the Italian response to the pandemic found that NPIs were successful in reducing SARS-CoV-2 transmission by significantly decreasing the number of social contacts.^14^ COVID-19 vaccination allowed a relaxation of restrictions and the progressive resumption of pre-pandemic social contacts.^15^ However, NPIs had significant effects on the transmission of several other respiratory viruses beyond SARS-CoV-2,^13^ including RSV.^16^

Modelling studies showed that large reductions in RSV transmission stemming from the implementation of NPIs would lead to an accumulation of susceptible individuals, resulting in large out-of-season RSV outbreaks when restrictions were relaxed.^17^, ^18^, ^19^, ^20^, ^21^, ^22^, ^23^ This predicted outcome was realised in Australia, Finland, Japan, and the UK, which experienced marked decreases in RSV case detection compared to previous winter seasons during the 2020–2021 season, followed by larger and/or earlier peaks in transmission in 2021–2022 when anti-COVID-19 measures were progressively relaxed.^24^, ^25^, ^26^, ^27^

Data from Lombardy in Northern Italy shows that RSV circulation followed a similar pattern, with a larger and earlier peak in hospitalisations in 2021–2022 experienced after extremely low levels of transmission in 2020–2021.^28^ It has been hypothesised that a gap in immunity caused by the implementation of NPIs played a key role in shaping the atypical 2021–2022 season,^26^^,^^29^ which is supported by the decreased levels of RSV antibody titres observed in Canada and the Netherlands.^30^^,^^31^

In this study, we use catalytic models applied to routinely collected hospitalisation discharge records, and epidemiological and virological surveillance data to investigate heterogeneities in RSV epidemiology and the impact of COVID-19 restrictions on the transmission intensity of RSV. Similar to proportional hazard models, catalytic models describe how the risk of an event per time unit changes over time, accounting for the expected duration of the time to event. Specifically, catalytic epidemic models can be used to estimate the accumulation of immunity from the age distribution of observed cases, by defining the force of infection (FOI) experienced at a given age a, the probability of remaining susceptible in the interval 0 to a−1, and the probability of acquiring infection at age a.^32^, ^33^, ^34^ By reconstructing the susceptibility profile of the population throughout the COVID-19 pandemic, we infer the extent to which changes in susceptibility and transmission explain the observed infection and hospitalisation patterns during the last four RSV seasons in Lombardy region.

## Methods

### Data

We analyse surveillance and hospitalisation data from 2018 to 2022. The 2019–2020 season was only partially affected by the NPIs implemented against COVID-19, which was first detected in Lombardy in February 2020.^13^ In contrast, the 2020–2021 season was characterised by restrictions imposed to counter the second COVID-19 wave in Italy and the emergence of the SARS-CoV-2 Alpha variant in early 2021.^13^^,^^35^ After the rollout of the national vaccination program against COVID-19, social distancing measures were progressively relaxed from the second half of 2021 onwards.^13^

We use age-stratified aggregated time-series of syndromic and virological surveillance data from the Italian influenza surveillance network (InfluNet),^36^ and aggregated hospital discharge data from Lombardy (∼10 M inhabitants)^37^ region provided by Regione Lombardia.^28^ InfluNet is a sentinel network of general practitioners (GPs) and paediatricians monitoring 2–4% of the approximately 10 million resident population of Lombardy and reporting weekly numbers of outpatients seeking care for influenza-like illness (ILI) along with the number of cases tested for respiratory viruses including RSV.^36^

The typical surveillance season spans weeks 46–17 of successive years. During the 2020–2021 season, surveillance was extended to include weeks 31–45 of 2021.^36^ The ILI and virological data are collected for four age-groups: 0–4, 5–14, 15–64, and 65+ years. The InfluNet data were used to estimate the number of RSV-attributable ILI cases per age-group and season, by multiplying the age-specific ILI incidence, the seasonal RSV test positivity rate, and the size of the Lombardy population^37^ [see the Supplementary Information (SI) and Pellegrinelli et al.^11^ for details].

These data were complemented with records provided by the official authorities at Regione Lombardia on the number of weekly hospital discharges due to RSV,^28^ made available for age-groups 0–6 months, 7–12 months, yearly age-groups up to age 80, and an aggregated group for individuals 81+ years.

### Catalytic model

We use a catalytic modelling approach^38^^,^^39^ to reconstruct the susceptibility profile of the Lombardy population to RSV primary infection and estimate the RSV FOI, λ(a,y), which is defined as the per-capita rate at which individuals of age a who had never experienced the infection in the past are infected in season y. We explore two scenarios, one accounting for maternally derived immunity (MDI) against infection, which we assumed to last for four months following birth^40^ (μ=1), and one with no MDI (μ=0). In the model, the probability of primary infection for a subject aged 0–12 months (a=0) in season y is given by

(1) $$P_{0}^{y,1}=\left(1-e^{-\lambda \left(0,y\right)}\right)\left(1-\mu /3\right)$$

Denoting ${\bar{P}}_{0}^{y,1}=1-P_{0}^{y,1}$ the probability of escaping primary infection at age a=0 in season y, the probability of primary infection for an individual of age a≥1 in season y is given by

(2) $$P_{a}^{y,1}={\bar{P}}_{0}^{y-a,1}\left(e^{-\sum_{i=1}^{a-1}\lambda \left(i,y-a+i\right)}\right)\left(1-e^{-\lambda \left(a,y\right)}\right)$$

where ${\bar{P}}_{0}^{y-a,1}\left(e^{-\sum_{i=1}^{a-1}\lambda \left(i,y-a+i\right)}\right)$ denotes the probability of escaping infection up to season y and $\left(1-e^{-\lambda \left(a,y\right)}\right)$ represents the probability of infection in season y (see SI for details). Similarly, the probability of post-primary RSV infection by an individual of age a≥1 in season y is defined by

(3) $$P_{a}^{y,2}=\left(1-{\bar{P}}_{0}^{\left(y-a\right),1}\left(e^{-\sum_{i=1}^{a-1}\lambda \left(i,y-a+i\right)}\right)\right)\left(1-e^{-s\lambda \left(a,y\right)}\right)$$

where parameter s= 0.77^10^ reflects the reduced susceptibility of individuals who have already experienced a first RSV infection.^9^^,^^10^ We computed the probability of RSV infection as the sum of the probabilities of primary and post-primary infection. For each season, we derived the expected number of RSV infections reporting ILI symptoms and the expected number of RSV hospitalisations, by estimating age-specific ascertainment and hospitalisation rates from surveillance data. The RSV test positivity ratio across different age-groups was also estimated making use of available virological records. We assumed that the probabilities of hospitalisation $\left(\rho^{Hosp}\right)$ and of reporting to surveillance given an RSV infection $\left(\rho^{Surv}\right)$, and the test positive ratio $\left(\rho^{V}\right)$ could all vary across age-groups but remained fixed throughout the considered seasons.

At the start of each season y, the initial epidemiological conditions can be quantified in terms of the proportion of individuals age a who have previously had RSV:

(4) $$\delta_{a,y}^{BEFORE}=\left(1-{\bar{P}}_{0}^{\left(y-a\right),1}\left(e^{-\sum_{i=1}^{a-1}\lambda \left(i,y-a+i\right)}\right)\right)$$

and the overall proportion of the population naïve to RSV:

(5) $${Naive}_{y}^{BEFORE}=1-\frac{\sum_{a}\delta_{a,y}^{BEFORE}\times N_{a}^{y}}{\sum_{a}N_{a}^{y}}$$

where $N_{a}^{y}$ is the population size at age a in season y. The immunity gap ($\Delta_{y}$) in any given season y can be computed as the difference in proportion of the RSV naïve population in that season compared to the pre-pandemic season, 2018–2019.

(6) $$\Delta_{y}={{Naive}_{y}^{BEFORE}-Naive}_{2018-2019}^{BEFORE}$$

We explored potential heterogeneities in exposure to the infection experienced by individuals at different ages and across seasons by estimating age-specific FOI values

(7) $$\lambda \left(a,y\right)=\left\{ \begin{array}{c} \lambda_{y}^{C},a\leq 4\\ \lambda_{y}^{Y},4<a\leq 14\\ \lambda_{y}^{A},a>14 \end{array}\right.$$

and testing 4 different model variants: (A) $\lambda_{y}^{C}=\lambda_{y}^{Y}=\lambda_{y}^{A}$; (B) $\left(\lambda_{y}^{C}=\lambda_{y}^{Y}\right)\neq \lambda_{y}^{A}$; (C) $\lambda_{y}^{C}\neq \left(\lambda_{y}^{Y}=\lambda_{y}^{A}\right)$; (D) $\lambda_{y}^{C}\neq \lambda_{y}^{Y}\neq \lambda_{y}^{A}$ (see Table 1 for details). In the model, λ(a,y) represents the FOI experienced by individuals with age a during the season y, which is assumed to be proportional to the cumulative number of RSV-attributable ILI cases identified during the season and an age- and season-specific per-capita transmission rate. We modelled the number of hospitalisations and RSV-attributable ILI cases with Poisson distributions, their age distributions with multinomial distributions, and the RSV testing data using a binomial distribution.

**Table 1:** Model descriptions and deviance information criterion.

	FOI assumption	Description	DIC
Model A	$\lambda_{y}^{C}=\lambda_{y}^{Y}=\lambda_{y}^{A}$	Age-constant and time-varying FOI.	18,165
Model B	$\left(\lambda_{y}^{C}=\lambda_{y}^{Y}\right)\neq \lambda_{y}^{A}$	Age- and time-varying FOI, with the assumption that FOI among 5–14-year-olds is equal to the FOI for 0–4-year-olds.	15,139
Model C	$\lambda_{y}^{C}\neq \left(\lambda_{y}^{Y}=\lambda_{y}^{A}\right)$	Age- and time-varying FOI, with the assumption that FOI among 5–14-year-olds is equal to the FOI for those 15-years-old and older.	8,195
Model D	$\lambda_{y}^{C}\neq \lambda_{y}^{Y}\neq \lambda_{y}^{A}$	Age- and time-varying FOI where the FOI across the three age-groups is allowed to freely vary.	6,088

95% exact binomial confidence intervals are shown for the RSV tests.

RSV, respiratory syncytial virus; ILI, influenza-like illness; CI, confidence interval.

DIC, deviance information criterion; FOI, force of infection.

Each model variant was calibrated to the observed seasonal hospital discharge and RSV-attributable ILI data using the Markov Chain Monte Carlo (MCMC) Bayesian inferential framework in Stan^41^ using the No-U-Turn Sampler (NUTS). For each model variant, four independent chains with randomised starting points were run for 20,000 iterations each, with 10,000 iterations used for warmup. The prior distributions were informed by published estimates^8^^,^^42^, ^43^, ^44^, ^45^, ^46^ (Supplementary Tables S1–S4). Model selection was conducted according to the deviance information criterion (DIC), a metric that is used for model selection by accounting for both the goodness of fit and the model complexity (in terms of the number of estimated parameters).^47^^,^^48^ We report the mean and 95% credible intervals (CrI) of the posterior distribution of the parameters. Model estimates of the immunity gap in the 2021–2022 and 2022–2023 seasons were computed. The estimate for 2022–2023 made use of 2020 mortality rates as obtained from the Italian National Institute of Statistics.^49^ A description of all model variables may be found in Supplementary Table S5.

### Sensitivity analyses

Beyond testing the impact of assuming no MDI (μ=0), we explored how model estimates were affected by a hypothetical increase of 25% and 50% in the probability of reporting RSV ($\rho^{Surv}$) between 2019 and 2022, as a result of increased testing during the pandemic (Table 2).

**Table 2:** Number of hospitalisations, RSV-attributable ILI cases, and RSV test positivity per season (total across all age-groups).

Season	Hospital discharges	RSV-attributable ILI cases	Positive tests/Total tests (%, 95% CI)
2018–2019	2,288	195,590	41/213 (19.25, 14.18–25.19%)
2019–2020	1,944	70,512	48/631 (7.61, 5.66–9.96%)
2020–2021	53	0	0/448 (0.00, 0.00–0.82%)
2021–2022	2,745	152,831	202/1121 (18.02, 15.81–20.40%)

95% exact binomial confidence intervals are shown for the RSV tests.

RSV, respiratory syncytial virus; ILI, influenza-like illness; CI, confidence interval.

DIC, deviance information criterion; FOI, force of infection.

Moreover, although results of a cohort study in Kilifi, Kenya highlighted that potential differences in disease severity after first and subsequent infection events were not significant when controlling for age,^8^ we explored the potential effect of considering a reduced probability of hospitalisation for post-primary RSV infections.

Full details on the model equations, variants, parameter prior distributions, and sensitivity analyses are available in the SI Methods section.

### Ethics

Ethical clearance and written informed consent were not required. The modelling study uses only aggregated counts and incidence records.

### Role of the funding source

None of the study funders were involved in the study design, data analysis, or writing of the manuscript.

## Results

### Data analysis

**Fig. 1:**
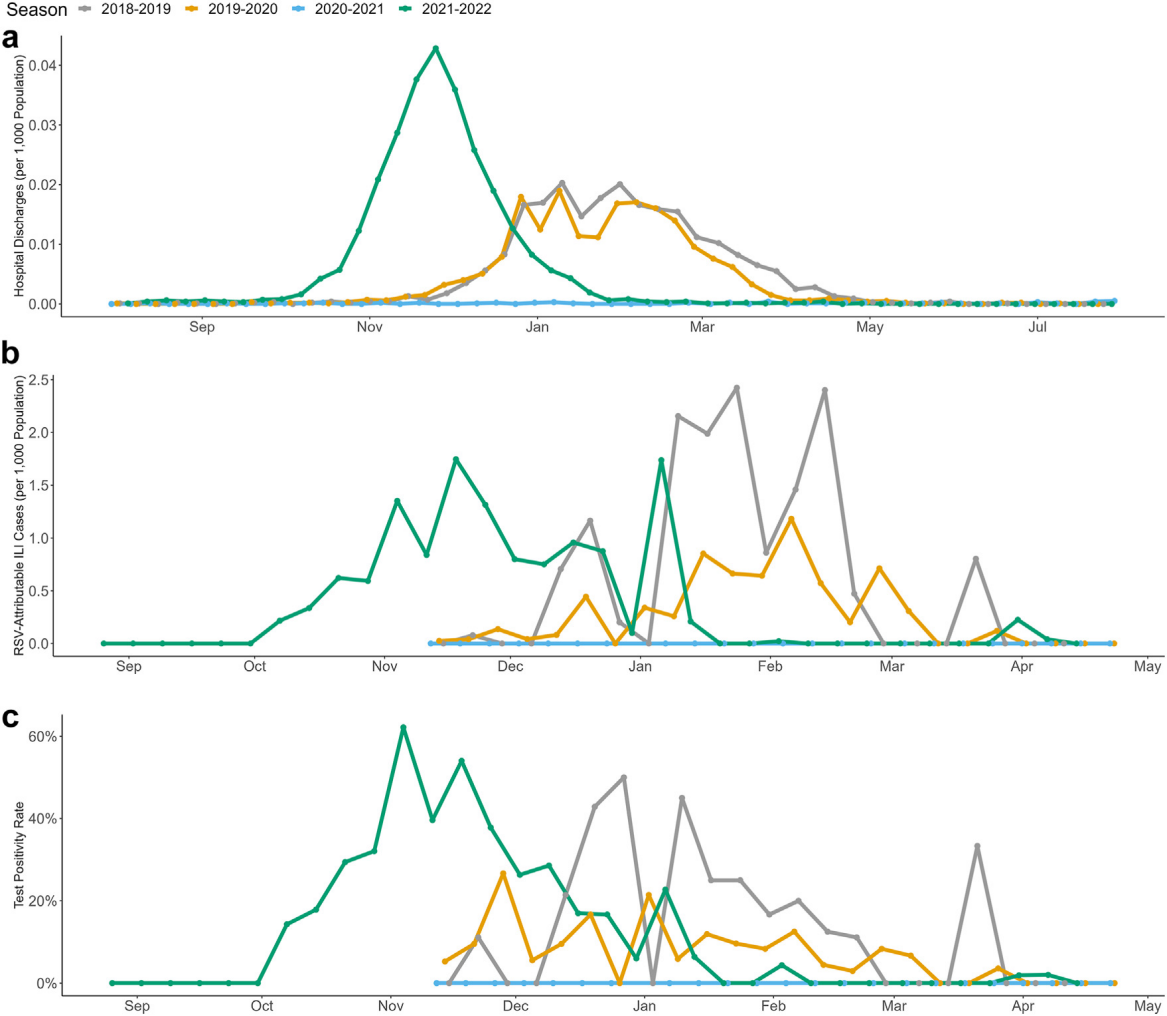
**Weekly RSV data from 2018–2019 to 2021–2022.** Panels show: (a) hospital discharges, (b) average reconstructed RSV-attributable ILIs, and (c) RSV test positivity rates. RSV, respiratory syncytial virus; ILI, influenza-like illness.

While the number of hospitalisations caused by RSV remained stable during the 2018–2019 and 2019–2020 seasons, the impact of the NPIs implemented against COVID-19 (which started in February 2020)^13^ was already visible in the number of RSV-attributable ILI cases and RSV positivity rates recorded in the 2019–2020 season (Fig. 1b and c).^13^ As shown in Table 1, there were fewer RSV-attributable ILI cases in 2019–2020 compared to 2018–2019, despite the larger number of tests performed (631 tests in 2019–2020 compared to 213 in 2018–2019).

No RSV-attributable ILI cases were recorded in 2020–2021, as all 448 tests performed during the season were negative. Moreover, there were only 53 hospitalisations, representing a 97.7% decrease from the 2,288 hospitalisations recorded in 2018–2019.

Fig. 1a shows that the 2021–2022 season was characterised by an atypical earlier and larger peak in hospitalisations compared to previous seasons. Interestingly, the larger number of hospitalisations was accompanied by fewer RSV-attributable ILI cases (152,831 in 2021–2022 versus 195,590 in 2018–2019 overall, of which 54,870 and 51,658 were in 0–4 year-olds, respectively) (Fig. 1b) despite similar test positivity rates overall (18.02% [95% CI: 15.81–20.40%] in 2021–2022 versus 19.25% [95% CI: 14.18–25.19%] in 2018–2019) and among 0–4 year olds (35.01% [95% CI: 30.54–39.69%] in 2021–2022 versus 39.62% [95% CI: 26.45–54.00%] in 2018–2019).

### Model results

**Fig. 2:**
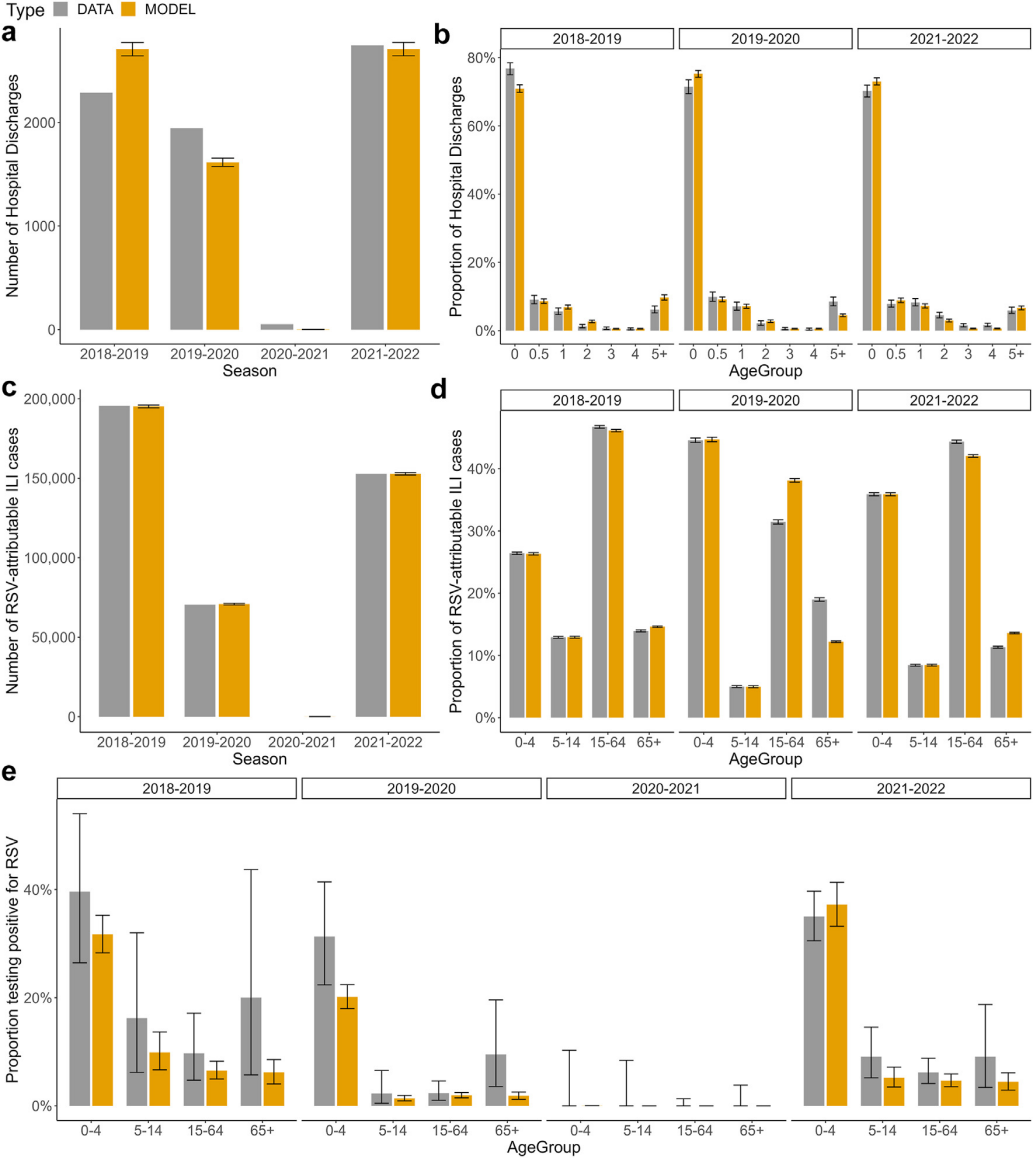
**Summary of model fit to data.** Observed and modelled (a) number of hospital discharges associated with RSV, (b) age-distribution of hospital discharges (age 0 refers to 0–6-month-olds and age 0.5 refers to 7–12-month-olds), (c) number of RSV-attributable ILI cases, (d) age-distribution of RSV-attributable ILI cases, and (e) RSV test positivity ratio. In all panels, error bars represent the 95% exact binomial CIs (where applicable) and the 95% CrIs for the model estimates. RSV, respiratory syncytial virus; ILI, influenza-like illness; CIs, confidence intervals; CrIs, credible intervals.

**Supplementary Figure S1:**
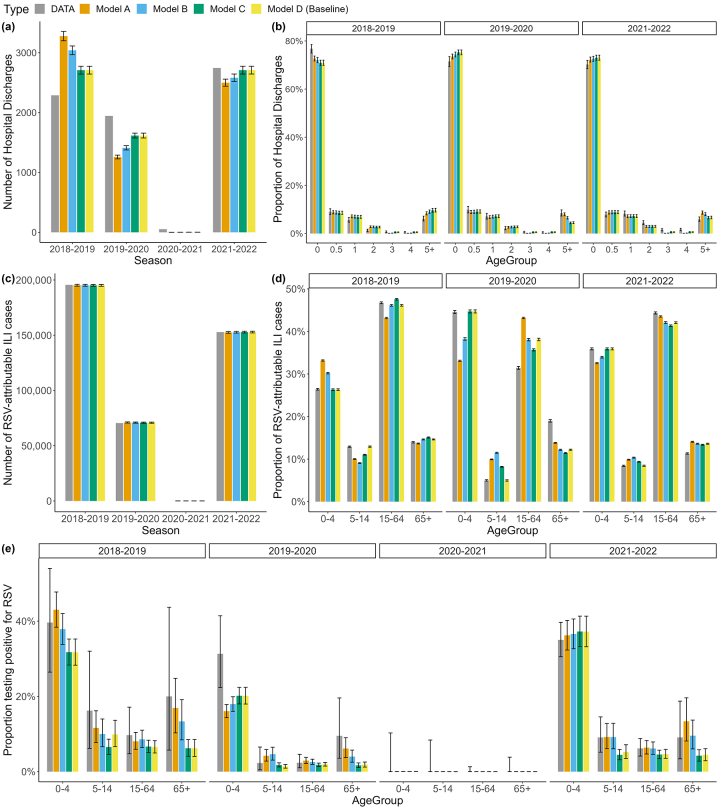
**Summary of model fit to data for scenarios with MDI.** Observed and estimated (a) number of hospital discharges associated with RSV, (b) age-distribution of hospital discharges (age 0 refers to 0–6-month-olds and age 0.5 refers to 7–12-month-olds), (c) number of RSV-attributable ILI cases, (d) age-distribution of RSV-attributable ILI cases, and (e) RSV test positivity ratio. In all panels, error bars represent the 95% exact binomial CIs around the data (where applicable) and the 95% CrIs for the model estimates. MDI, maternally derived immunity; RSV, respiratory syncytial virus; ILI, influenza-like illness; CIs, confidence intervals; CrIs, credible intervals.

The model allowing the FOI to vary across the three age-groups (model D) was preferred according to the DIC (Table 2). Hereafter, we present the estimates obtained with model D and refer to it as the baseline model. Fig. 2 shows that the baseline model can reproduce the number and age-distribution of hospital discharges associated with RSV, the number and age-distribution of RSV-attributable ILI cases, and the RSV test positivity ratio per age-group. The model fit and parameter estimates of other model variants are presented in Supplementary Figure S1 and Supplementary Table S6, respectively.

**Fig. 3:**
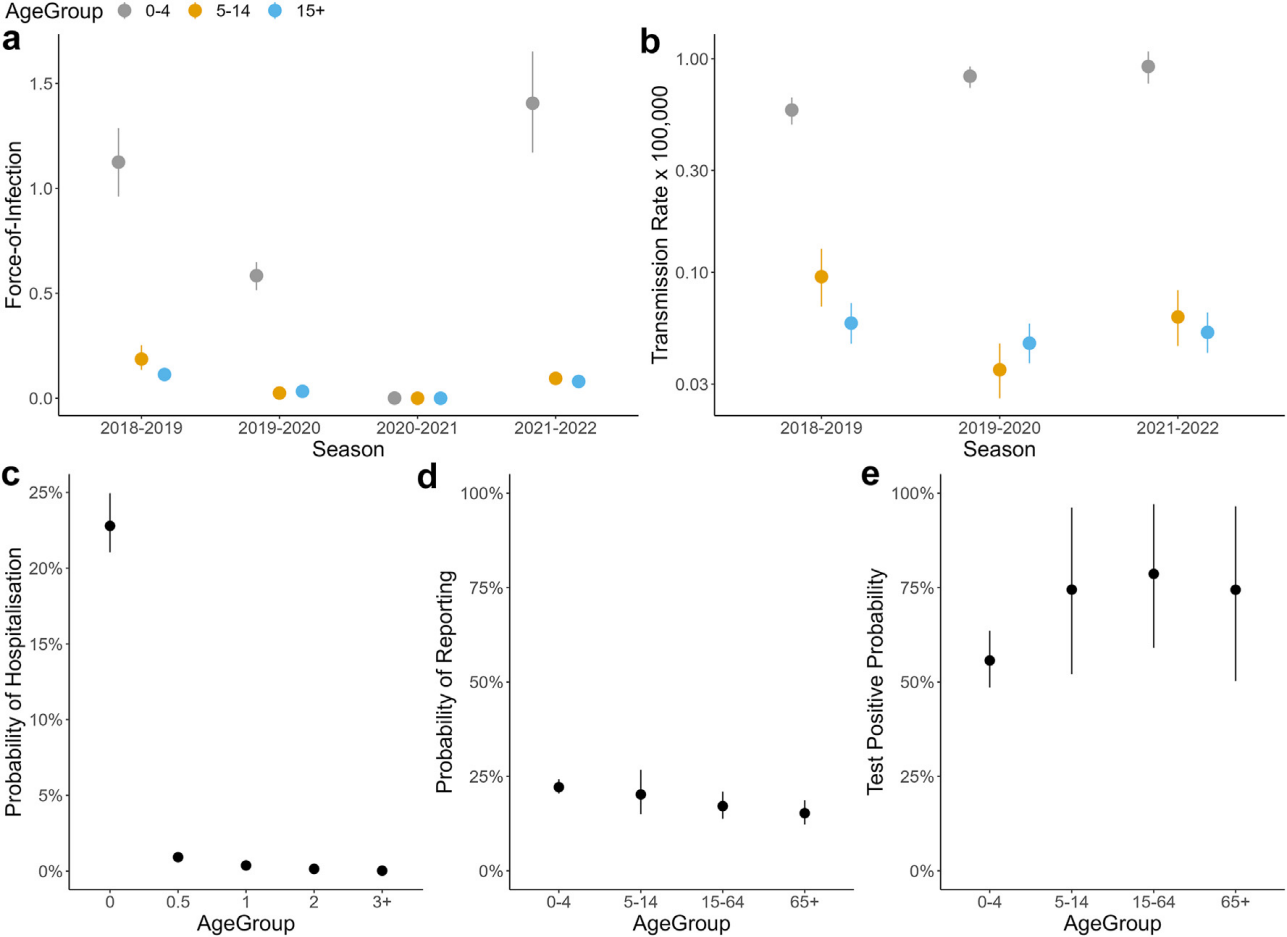
**Model estimates.** Estimated (a) FOI for the three age-groups 0–4, 5–14, and 15+ years, (b) per-capita RSV transmission rates, as estimated by dividing the FOI model estimates by the cumulative number of RSV-attributable ILI cases reported each season (y-axis on a log-scale); and probability of (c) hospitalisation, (d) a case being symptomatic and reported to surveillance, and (e) test positive ratio associated with RSV. In all panels, points represent the mean and error bars represent 95% CrIs. FOI, force of infection; RSV, respiratory syncytial virus; ILI, influenza-like illness; CrIs, credible intervals.

The parameter estimates show large reductions in the FOI across all age-groups in 2019–2020, the season when the COVID-19 pandemic emerged, followed by a nearly complete suppression of RSV transmission in 2020–2021, and a substantially larger FOI in 2021–2022, comparable in size to the pre-COVID season 2018–2019 (Fig. 3a). Among those 0–4 years, the FOI estimates in 2021–2022 are on average 0.28 (95% CrI: 0.20–0.37) higher than those in the pre-COVID season. While the FOI among those 0–4 years decreased from 2018–2019 to 2019–2020, our results suggest that in the same period the per-capita RSV transmission rate increased (from 0.58 [95% CrI: 0.49–0.66] × 100,000 season^−1^ to 0.83 [95% CrI: 0.73–0.92] × 100,000 season^−1^) (Fig. 3b). In contrast, the per-capita RSV transmission rate associated with individuals aged 5–14 years dropped from 0.10 (95% CrI: 0.07–0.13) × 100,000 season^−1^ in 2018–2019 to 0.04 (95% CrI: 0.03–0.05) × 100,000 season^−1^ in 2019–2020. A less evident reduction was found among those 15+ years: from 0.06 (95% CrI: 0.05–0.07) × 100,000 season^−1^ in 2018–2019 to 0.05 (95% CrI: 0.04–0.06) × 100,000 season^−1^ in 2019–2020.

Our estimates show that both the FOI and transmission rates in 2021–2022 among 5–14 and 15+ years age-groups remained below the pre-COVID levels, suggesting that spontaneous behavioural changes and COVID-19 related measures likely contributed to a reduction in RSV circulation in 2021–2022 compared to the pre-COVID period.^13^

We estimate a 22.8% (95% CrI: 21.1–24.9%) probability of hospitalisation among RSV infected individuals aged 0–6 months, which decreases to almost zero in the 3+ years age-group (Fig. 3c and Supplementary Table S5). Our estimates suggest that the probability of reporting ILI symptoms to surveillance is similar across age-groups, ranging from 22.1% (95% CrI: 20.5–24.2%) among 0–4 year-olds to 15.3% (95% CrI: 12.3–18.7%) among 65+ year-olds (Fig. 3d).

On average, the RSV test positive ratio was higher in individuals 5+ years (75.9% [95% CrI: 62.6–88.6%]) than in children aged 0–4 years (55.7% [95% CrI: 48.6–63.6%]), possibly reflecting higher rates of respiratory infections beyond RSV, in young children (Fig. 3e).

**Fig. 4:**
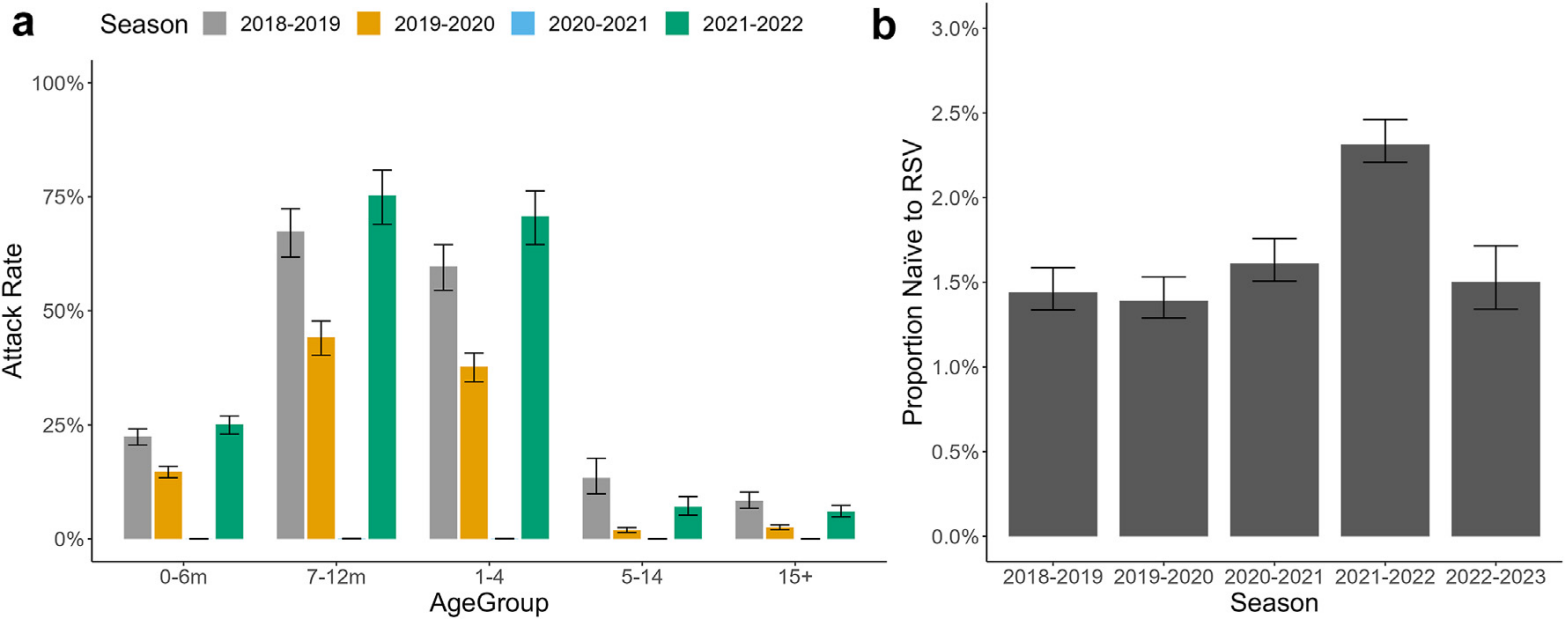
**Attack rates and estimates of the proportion of the population naïve to RSV.** Estimated (a) RSV attack rates by age-group per season (bars represent the mean, and error bars the 95% CrIs) and (b) proportion of Lombardy population naïve to RSV infection at the start of each season. RSV, respiratory syncytial virus; CrIs, credible intervals.

We estimate that the RSV infection attack rate among 1–4 year-olds increased from 59.7% (95% CrI: 54.5–64.5%) in 2018–2019 to 70.7% (95% CrI: 64.5–76.3%) in 2021–2022 (Fig. 4a). In older individuals, lower attack rates were found compared to the pre-COVID season. This is likely the result of an immunity gap led by the reduced circulation of RSV caused by the NPIs implemented to counter the spread of SARS-CoV-2. In fact, we found an immunity gap of 0.87% (95% CrI: 0.87–0.88%), which represents a 60.8% (95% CrI: 55.2–65.4%) increase in the proportion of the Lombardy population naïve to RSV at the start of the 2021–2022 season (2.3% [95% CrI: 2.2–2.5%]) compared to 2018–2019 (1.4% [95% CrI: 1.3–1.6%]; Fig. 4b). The proportion of the population 1–5 years (young children) who were RSV naïve increased from 15.6% (95% CrI: 13.6–18.1%) in 2018–2019 to 40.5% (95% CrI: 38.5–43.0%) in 2021–2022. This represents an immunity gap of 24.97% (95% CrI: 24.79–25.14%) among individuals 1–5 years.

We estimate 1.5% (95% CrI: 1.3–1.7%) of the population naïve to RSV at the start of the following 2022–2023 season, which compared to the 2018–2019 estimate (1.4% [95% CrI: 1.3–1.6%]) suggests that the immunity gap has almost completely been filled after the resurgent 2021–2022 season.

### Sensitivity analyses

**Supplementary Figure S2:**
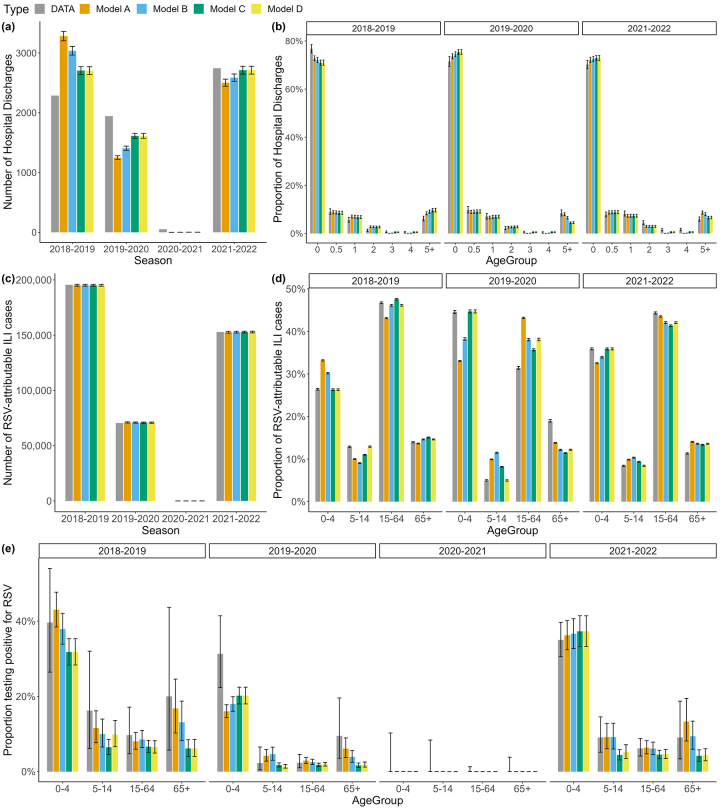
**Summary of model fit to data for scenarios without MDI.** Observed and estimated (a) number of hospital discharges associated with RSV, (b) age-distribution of hospital discharges (age 0 refers to 0–6-month-olds and age 0.5 refers to 7–12-month-olds), (c) number of RSV-attributable ILI cases, (d) age-distribution of RSV-attributable ILI cases, and (e) RSV test positivity ratio. In all panels, error bars represent the 95% exact binomial CIs around the data (where applicable) and the 95% CrIs for the model estimates. MDI, maternally derived immunity; RSV, respiratory syncytial virus; ILI, influenza-like illness; CIs, confidence intervals; CrIs, credible intervals.

**Supplementary Figure S3:**
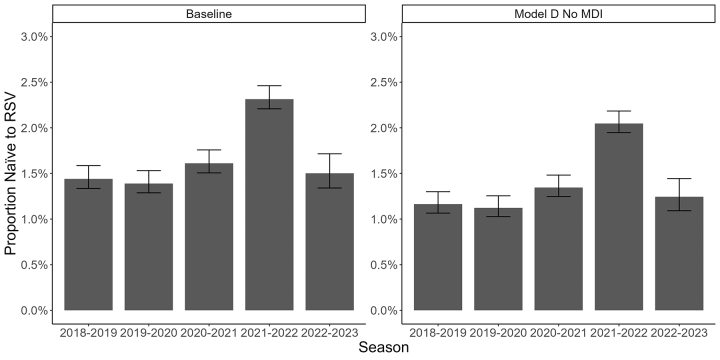
**Proportion naïve to RSV over time**. Estimates obtained with the baseline model (left), and the sensitivity analysis with Model D and no MDI (right). MDI, maternally derived immunity; RSV, respiratory syncytial virus.

Using model D with no MDI, we estimated that the proportion of the population naïve to RSV increased from 1.2% (95% CrI: 1.1–1.3%) in 2018–2019 to 2.0% (95% CrI: 1.9–2.2%) at the start of the 2021–2022 season, therefore suggesting an immunity gap of 0.88% (95% CrI: 0.88–0.89%). Due to the absence of MDI, we estimated a larger number of RSV infections among individuals aged 0–6 months and hence a reduced estimated probability of hospitalisation in this age-group (7.62% [95% CrI: 7.03–8.35%]). Model fit, parameter estimates, and immunity gap results assuming the absence of MDI are presented in Supplementary Figures S2, Supplementary Table S7, and Supplementary Figure S3, respectively.

**Supplementary Figure S4:**
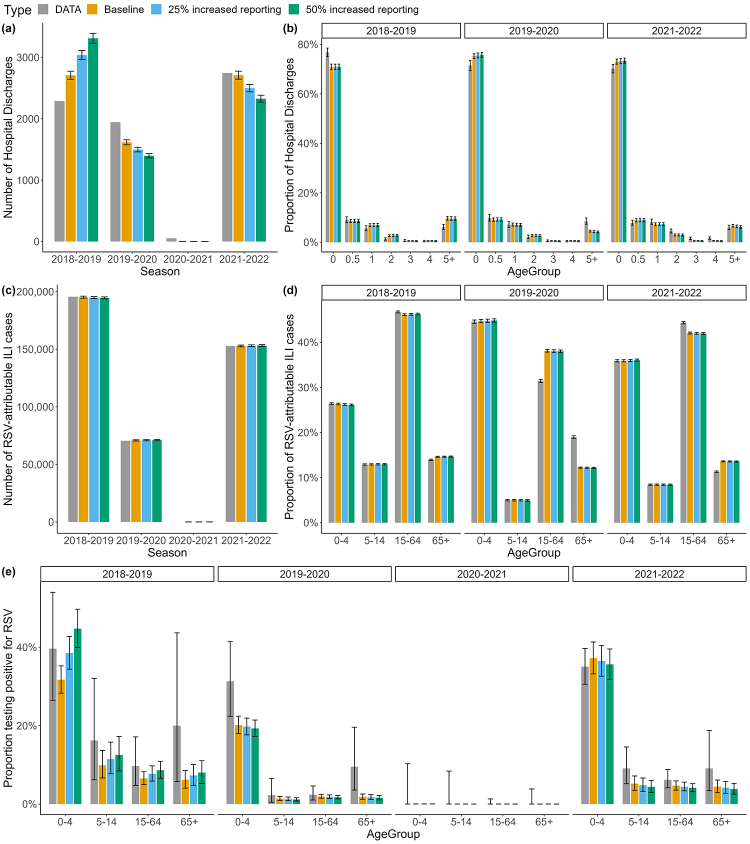
**Summary of model fit to data for ahypothetical25% and 50% increase in reporting during pandemic affected seasons versus baseline.** Observed and estimated (a) number of hospital discharges associated with RSV, (b) age-distribution of hospital discharges (age 0 refers to 0–6-month-olds and age 0.5 refers to 7–12-month-olds), (c) number of RSV-attributable ILI cases, (d) age-distribution of RSV-attributable ILI cases, and (e) RSV test positivity ratio. In all panels, error bars represent the 95% exact binomial CIs around the data (where applicable) and the 95% CrIs for the model estimates. RSV, respiratory syncytial virus; ILI, influenza-like illness; CIs, confidence intervals; CrIs, credible intervals.

**Supplementary Figure S5:**
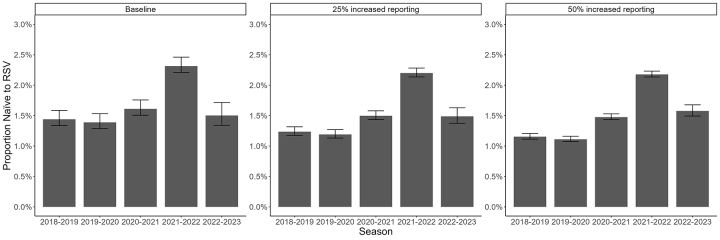
**Estimated proportion naïve to RSV over time.** Estimates obtained with the baseline model (left), and the two sensitivity analyses with 25% increased reporting (central) and 50% increased reporting (right). RSV, respiratory syncytial virus.

**Supplementary Figure S6:**
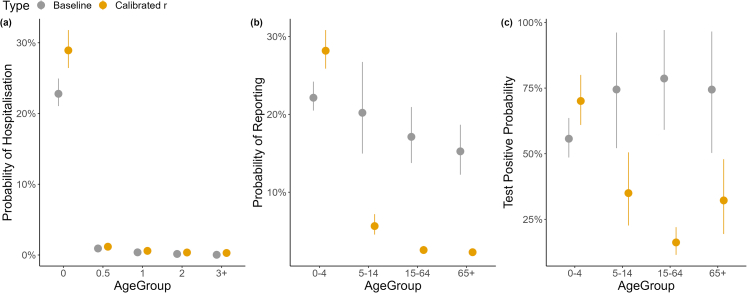
**Parameter estimates for the baseline and calibrated**r (reduced probability of hospitalisation for post-primary infections) **models.** (a) Probability of hospitalisation, (b) probability of reporting to surveillance, and (c) Test positive probability.

**Supplementary Figure S7:**
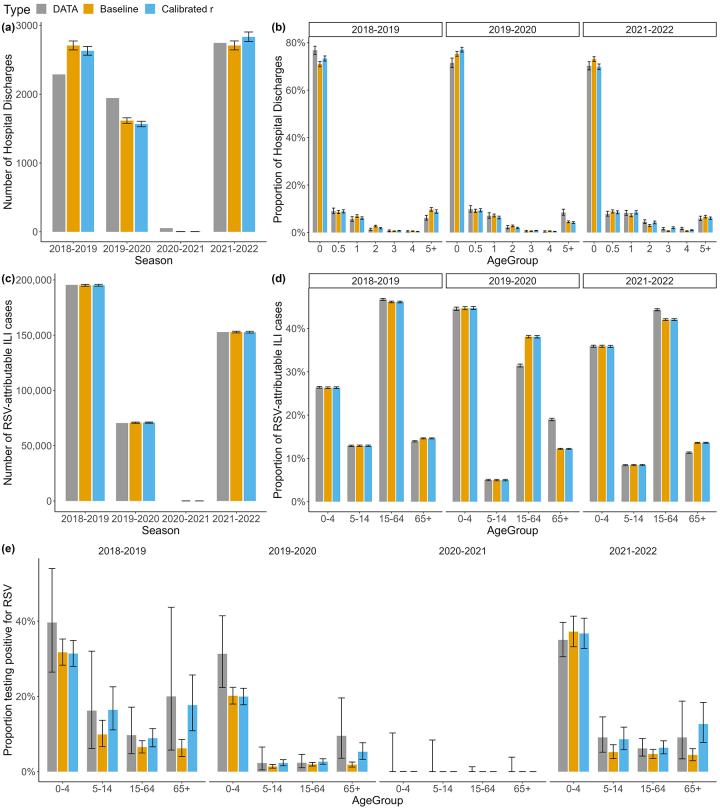
**Summary of model fit to data for calibrated**r**(reduced probability of hospitalisation for post-primary infections)versus baseline model.** Observed and estimated (a) number of hospital discharges associated with RSV, (b) age-distribution of hospital discharges (age 0 refers to 0–6-month-olds and age 0.5 refers to 7–12-month-olds), (c) number of RSV-attributable ILI cases, (d) age-distribution of RSV-attributable ILI cases, and (e) RSV test positivity ratio. In all panels, error bars represent the 95% exact binomial CIs around the data (where applicable) and the 95% CrIs for the model estimates. RSV, respiratory syncytial virus; ILI, influenza-like illness; CIs, confidence intervals; CrIs, credible intervals.

**Supplementary Figure S8:**
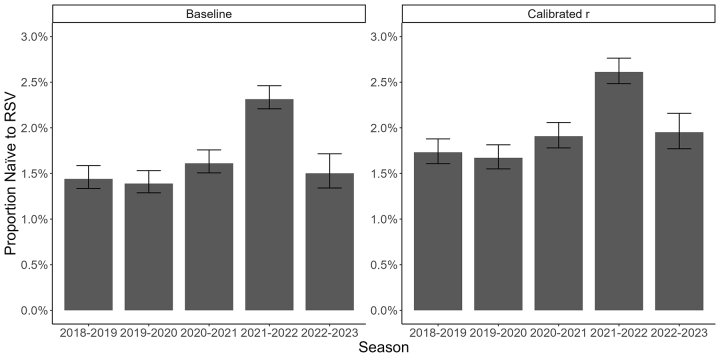
**Proportion naïve to RSV over time.** Estimates obtained with the baseline model (left) and the sensitivity analysis with a reduced probability of hospitalisation for post-primary infections (right). RSV, respiratory syncytial virus.

A slightly larger immunity gap of 0.96% (95% CrI: 0.96–0.97%) and 1.02% (95% CrI: 1.02–1.03%) was estimated when assuming a 25% and 50% increase in $\rho^{Surv}$ for seasons 2019–2022, respectively. When a reduced probability of hospitalisation for post-primary infections was considered, the proportion of the population naïve to RSV was estimated to increase from 1.7% (95% CrI: 1.6–1.9%) at the start of 2018–2019 to 2.6% (95% CrI: 2.5–2.8%) in 2021–2022, with an immunity gap of 0.88% (95% CrI: 0.87–0.89%) that is consistent with our baseline estimates. Full results of all sensitivity analyses performed are shown in the SI Results section (Supplementary Figures S4 and S5 and Supplementary Table S8 for scenarios with increased reporting, and Supplementary Figures S6–S8 and Supplementary Table S9 for the scenario with reduced probability of hospitalisation for post-primary infections).

## Discussion

In this study, we analysed RSV surveillance and hospitalisation data from Lombardy region in Northern Italy throughout the COVID-19 pandemic, where an absent 2020–2021 season followed by an atypical larger and earlier peak in RSV hospitalisations in 2021–2022 was observed. This is in line with other reports from Japan, Finland, Australia, and the UK.^24^, ^25^, ^26^, ^27^ Previous studies hypothesised that this resurgence may result from an immunity gap caused by reduced RSV exposure stemming from the implementation of NPIs.^26^ This hypothesised link between the immunity gap generated from the implementation of NPIs and the resurgence of RSV was forecasted by prospective modelling studies applied to data from Norway, USA, UK, South Africa [preprint], Hong Kong, and Japan.^17^, ^18^, ^19^, ^20^, ^21^, ^22^, ^23^ A solid quantitative assessment of such immunity gap from observed data represents a key priority for RSV research and preparedness planning.^26^^,^^29^

To address this important issue, we developed a modelling study based on the use of catalytic models, which allowed reconstruction of the susceptibility profile of the population. The model was informed by hospital discharge records, and epidemiological and virological surveillance data from four transmission seasons spanning the years 2018–2022.

Our estimates of the proportion of individuals who previously experienced RSV infection in the pre-COVID 2018–2019 season (45.0% [95% CrI: 41.2–48.3%] of 1-year-olds, and 82.0% [95% CrI: 77.5–85.7%] of 2-year-olds), are comparable to the results obtained in a serological study conducted in the Netherlands before the COVID-19 pandemic, which found that 44.1% of 1-year-olds and 84.6% of 2-year-olds had experienced primary infection.^42^

By accounting for heterogeneity in RSV circulation across age-groups over time, we inferred variations in the impact of the NPIs implemented against COVID-19 on the risk of infection experienced at different ages. Our estimates suggest that the COVID-19 restrictions likely caused a marked reduction of potentially infectious contacts in individuals aged 5–14 years, and to a lower extent in individuals aged 15+ years. This is at least partially ascribable to school closures widely implemented in the first two years of the pandemic.^13^ However, results obtained for the 2021–2022 season show that, after restrictions were relaxed,^13^ both the FOI and transmission rates among individuals aged 5–14 years remained below pre-COVID levels. On the other hand, the RSV FOI for children aged 0–4 years was larger in 2021–2022 compared to 2018–2019. This increase could be linked to reductions in RSV exposure for expectant mothers during pregnancy which may have affected the extent and proportion of maternally derived immunity acquired at birth by children who were born during the pandemic.^30^^,^^31^

The model developed in this analysis has limitations. For instance, we assumed a fixed age-independent reduction in susceptibility for post-primary infections (s = 0.77).^10^ We also did not explicitly consider the progressive waning of MDI nor include potential reductions in the duration of MDI inherited by children born during the pandemic resulting from reduced RSV exposure of mothers. Possible differences in the epidemiology of RSV subgroup A and B were not considered either. Nonetheless, our estimates of the immunity gap determined by COVID-19 restrictions are robust with respect to the assumptions we made on the potential contribution of maternal immunity in protecting against the infection, the probability of hospitalisation for post-primary infections, and potential changes in the reporting of RSV cases determined by increased testing during the pandemic.

In sum, our modelling analysis clearly shows that the unexpected increase in RSV hospitalisations observed in 2021–2022 could be at least partially ascribed to a higher incidence of primary RSV infection among young individuals. This is well reflected by the 60% increase we estimated for the proportion of individuals without prior RSV exposure.

More in general, this study shows how data fusion modelling approaches linking different streams of surveillance data can provide new insight into RSV epidemiology, its transmission patterns, and the role that COVID-19 restrictions had on the changes in immunity and observed epidemic dynamics. Notably, the methods developed in this study can be used in the future to continue to monitor RSV circulation, as well as the transmission intensity of other infectious diseases using routinely collected surveillance data from around the world.

## Contributors

The study was conceptualised by ID and PP. HJA, ID, EP, LP, GP, GMS, PP, and MM curated the data. HJA performed the formal analysis. Funding was acquired by ID, SM, and PP. HJA, ID, PP, FM, DC, and MT designed the methodology. Software was developed by HJA. The underlying data was verified by MT, DC, and GP. HJA, ID, and PP generated the visuals. The original draft of the manuscript was written by HJA, ID, and PP and was reviewed and edited by ID, EP, SM, FM, PP, SB, DC, and MT. The project was supervised by ID, SM, and PP. All authors had full access to the study data, have reviewed and approved the final version of the manuscript.

## Data sharing statement

Model code and aggregated data are available online at https://github.com/hadrianang/RSV-Lombardy. More granular time series data may be made upon reasonable request to the corresponding authors.

## Declaration of interests

The authors declare no competing interests.
