# Supplementary material for: Reconstructing the impact of COVID-19 on the immunity gap and transmission of respiratory syncytial virus in Lombardy, Italy
Source: eBioMedicine. 2023 Aug 9;95:104745. doi: 10.1016/j.ebiom.2023.104745 (PMC10432612; doi:10.1016/j.ebiom.2023.104745)
Supplement: Supplementary Information [file mmc1.docx]

Supplementary Information

Contents

[Methods 2](#_Toc142046120)

[Mathematical model 2](#_Toc142046121)

[Hospital discharges 3](#_Toc142046122)

[RSV-attributable ILI 4](#_Toc142046123)

[Virological testing 5](#_Toc142046124)

[Likelihood formulation 5](#_Toc142046125)

[Proportion Fully Susceptible to RSV 5](#_Toc142046126)

[Estimating Transmission Rates 6](#_Toc142046127)

[Immunity Profile Prior to 2022–2023 6](#_Toc142046128)

[Model Parameters 7](#_Toc142046129)

[Prior distribution for $\lambda$ 8](#_Toc142046130)

[Prior distribution for $\rho Hosp$, $\rho Surv$, $\rho V$ 9](#_Toc142046131)

[Model variables 9](#_Toc142046132)

[Model selection 11](#_Toc142046133)

[Sensitivity Analysis: Increased probability of reporting to surveillance ($\rho Surv$) during seasons affected by the pandemic 11](#_Toc142046134)

[Sensitivity Analysis: Calibrated factor $r$ for reduced probability of hospitalisation for post-primary RSV infections 11](#_Toc142046135)

[Results 12](#_Toc142046136)

[Parameter estimates and model fit – scenarios with MDI ($\mu$ = 1) 12](#_Toc142046137)

[Sensitivity Analysis: Scenarios without MDI ($\mu$ = 0) 13](#_Toc142046138)

[Sensitivity Analysis: Increased probability of reporting to surveillance ($\rho Surv$) during seasons affected by the pandemic 14](#_Toc142046139)

[Sensitivity Analysis: Calibrated factor $r$ for reduced probability of hospitalisation for post-primary RSV infections 16](#_Toc142046140)

[References 18](#_Toc142046141)

# **Methods**

## **Mathematical model**

We assumed that the force of infection (FOI) $\lambda\left( a,y \right)$ varies by age ($a$) and season ($y$) and is piecewise constant for three age-groups, 0-4, 5-14, and 15+ years as follows:

| $\lambda\left( a,y \right)=\left\{ \begin{aligned} &\lambda_{y}^{C}&, &a\leq4 \\ &\lambda_{y}^{Y}&, &4<a\leq14 \\ &\lambda_{y}^{A}&, &a>14 \end{aligned} \right.$ | (1) |
| --- | --- |

Seasons prior to the four season study period are assumed to have the same FOI as the first season in the study period, 2018–2019. Let $\mu\in\{0,1\}$ denote the degree of maternally derived immunity (MDI) such that if $\mu=1$, we assume that individuals are born with four months of complete MDI, while if $\mu=0$ there is no MDI. Assuming that the age of individuals 0-6 months is equally distributed, the probability of primary RSV infection for an individual age 0-6 months in season $y$ is given as

| $P_{0-6m}^{y,1}=\left( P_{0-6m}^{y,1} \vert0m<a<4m \right)p\left( 0m<a<4m \right)(1-\mu)+\left( P_{0-6m}^{y,1} \vert4m<a<6m \right)p\left( 4m<a<6m \right)$ | (2) |
| --- | --- |

where $\left( P_{0-6m}^{y,1} | 0m<a<4m \right)$ is the probability of primary infection given an individual is 0–4 months-old and $\left( P_{0-6m}^{y,1} | 4m<a<6m \right)$is the probability of primary infection given an individual is 4-6 months-old. From this, we have the following equations.

| $\left( P_{0-6m}^{y,1} \vert0m<a<4m \right)p\left( 0m<a<4m \right)(1-\mu)=\left( 1-e^{-\lambda\left( 0,y \right)} \right)\times2/3\times(1-\mu)$ $\left( P_{0-6m}^{y,1} \vert4m<a<6m \right)p\left( 4m<a<6m \right)=\left( 1-e^{-\lambda\left( 0,y \right)} \right)\times1/3$ $P_{0-6m}^{y,1}=\left( 1-e^{-\lambda\left( 0,y \right)} \right)(1-2\mu/3)$ | (3) |
| --- | --- |

The probability of primary infection for an individual 7–12 months in season $y$ is given by

| $P_{7-12m}^{y,1}=\left( 1-e^{-\lambda\left( 0,y \right)} \right)$ | (4) |
| --- | --- |

Since season $y$ is the first RSV season an individual younger than 1-years-old experiences, the probability of post-primary infection for individuals 0-6 months and 7-12 months is zero:

| $P_{0-6m}^{y,2}=P_{7-12m}^{y,2}=0$ | (5) |
| --- | --- |

Combining the probabilities of primary infection for those 0-6 and 7-12 months, the probability of primary infection by an individual in their first year of life in season $y$ $\left( P_{0}^{y,1} \right)$ is given by the following equation:

| $P_{0}^{y,1}=P_{0-6m}^{y,1}p\left( 0m<a<6m \right)+P_{7-12m}^{y,1}p(7m<a<12m)$ $P_{0}^{y,1}=\left( \left( 1-e^{-\lambda\left( 0,y \right)} \right)\left( 1-2\mu/3 \right) \right)(0.5)+\left( 1-e^{-\lambda\left( 0,y \right)} \right)\left( 0.5 \right)$ $P_{0}^{y,1}=\left( 1-e^{-\lambda\left( 0,y \right)} \right)(1-\mu/3)$ | (6) |
| --- | --- |

The probability that an individual in their first year of life in season $y$ escapes primary RSV infection ${(\bar{P}}_{0}^{y,1})$ is then given by

| $\bar{P}_{0}^{y,1}=1-\left( P_{0}^{y,1} \right)=1-\left( 1-e^{-\lambda\left( 0,y \right)} \right)(1-\mu/3)$ | (7) |
| --- | --- |

The probability of primary infection for an individual of age $a\geq1$ in season $y$ is given by

| $P_{a}^{y,1}=\bar{P}_{0}^{\left( y-a \right),1}\left( e^{-\sum_{i=1}^{a-1} \lambda\left( i, y-a+i \right)} \right)\left( 1-e^{-\lambda(a,y)} \right)$ | (8) |
| --- | --- |

where $\bar{P}_{0}^{\left( y-a \right),1}\left( e^{-\sum_{i=1}^{a-1} \lambda\left( i, y-a+i \right)} \right)$ denotes the probability of escaping infection across all previous seasons since birth and $\left( 1-e^{-\lambda(a,y)} \right)$ represents the probability of infection in season $y$. Similarly, the probability of post-primary RSV infection by an individual age $a\geq1$ in season $y$ is given by

| $P_{a}^{y,2}=\left( 1-\bar{P}_{0}^{\left( y-a \right),1}\left( e^{-\sum_{i=1}^{a-1} \lambda\left( i, y-a+i \right)} \right) \right)\left( 1-e^{-s\lambda(a,y)} \right)$ | (9) |
| --- | --- |

where $s$ denotes the reduced susceptibility to post-primary infections. The probability of RSV infection for an individual of age $a$ in season $y$ is then given as the sum of the probabilities of primary and post-primary infection.

| $P_{a}^{y}=P_{a}^{y,1}+P_{a}^{y,2}$ | (10) |
| --- | --- |

We define the average probability of an individual in age-group $g$ being infected by RSV in season $y$, $\pi_{g}^{y}$, as

| $\pi_{g}^{y}=\frac{\sum_{a\in g} P_{a}^{y}N_{a}^{y}}{\sum_{a\in g} N_{a}^{y}}$ | (11) |
| --- | --- |

where $N_{a}^{y}$ is the number of individuals of age $a$ in season $y$.

## **Hospital discharges**

From the data, we compute the total number of discharges per season $y$ in age-group $g$, $H_{g,y}$, by summing the weekly age-specific discharges $H_{g,w}$ for each week $w$ in season $y$.

| $H_{g,y}=\sum_{w\in y} H_{g,w}$ | (12) |
| --- | --- |

The expected number of hospitalisations in age-group $g$ in season $y$, $\Lambda_{g,y}^{Hosp}$, is given by

| $\Lambda_{g,y}^{Hosp}=\pi_{g}^{y}\rho_{m}^{Hosp}N_{g}^{y}$ | (13) |
| --- | --- |

where $\rho_{m}^{Hosp}$ is the probability of hospitalisation due to RSV in age-group $m$ (with $g\subseteq m$), and $N_{g}^{y}$ is the number of individuals in age-group $g$ in season $y$. We assume that the probability of hospitalisation remains constant in time but varies by age according to the following age-groups: 0-6 months, 7-12 months, 1-2 years, 2-3 years, and 3+ years. Since only yearly population data were available, we assumed an equal number of 0-6 and 7-12 month-olds. The distribution of hospitalisations by age-group, $\theta_{g,y}^{Hosp}$, is given by

| $\theta_{g,y}^{Hosp}=\frac{\Lambda_{g,y}^{Hosp}}{\sum_{k} \Lambda_{k,y}^{Hosp}}$ | (14) |
| --- | --- |

We assume that the total number of hospital discharges in season $y$ follows a Poisson distribution with mean equal to the modelled expected number of hospital discharges.

| $\sum_{k} H_{k,y}\sim\mathrm{Poisson}\left( \sum_{k} \Lambda_{k,y}^{Hosp} \right)$ | (15) |
| --- | --- |

Moreover, we assumed that the age-distribution of the hospital data in season $y$, $H_{y}$, followed a multinomial distribution,

| $H_{y}\sim\mathrm{Multinomial}\left( \sum_{k} H_{k,y}, \theta_{y}^{Hosp} \right)$ | (16) |
| --- | --- |

where the probabilities are given by $\theta_{y}^{Hosp}$, the modelled age-distribution of hospitalisations in season $y$. We excluded the 2020-2021 season from the modelling of the age-distribution due to the small number of hospitalisations (53).^1^

## **RSV-attributable ILI**

For each age-group $j$ and week $w$, InfluNet provides the reported weekly incidence of influenza-like illness (ILI) per 1 000 individuals, $I_{j,w}$, and the number of positive RSV tests $P_{j,w}$ among those tested $T_{j,w}$.^2^ For a given RSV season $y$, we reconstructed the number of RSV-attributable ILI cases for age-group $j$, $R_{j,y}$, as follows

| $R_{j,y}=\left\lfloor\left( \sum_{w\in y} I_{j,w} \right)\times\left( \frac{\sum_{w\in y} P_{j,w}}{\sum_{w\in y} T_{j,w}} \right)\times\frac{N_{j}^{y}}{1000} \right\rfloor$ | (17) |
| --- | --- |

where $N_{j}^{y}$ is the population size of age-group $j$ during season $y$. The expected number of RSV-attributable ILI cases in age-group $j$ during season $y$, $\Lambda_{j,y}^{Surv}$, was modelled as

| $\Lambda_{j,y}^{Surv}=\pi_{j}^{y}\rho_{j}^{Surv}N_{j}^{y}$ | (18) |
| --- | --- |

where $\rho_{j}^{Surv}$ represents the probability that an RSV infection in age-group $j$ is symptomatic and reported to surveillance, and $N_{j}^{y}$ is the population of age-group $j$ in season $y$. As done for the probability of hospitalisation, we assumed that the probability of reporting to surveillance varies by age but remains constant across seasons. The proportion of RSV-attributable ILI cases in age-group $j$ in season $y$, $\sigma_{j,y}^{Surv}$, is given by

| $\sigma_{j,y}^{Surv}=\frac{\Lambda_{j,y}^{Surv}}{\sum_{k} \Lambda_{k,y}^{Surv}}$ | (19) |
| --- | --- |

We model the total number of RSV-attributable ILI cases during season $y$ using a Poisson likelihood with mean equal to the modelled expected number of cases in the same age-group and season.

| $\sum_{k} R_{k,y}\sim\mathrm{Poisson}\left( \sum_{k} \Lambda_{k,y}^{Surv} \right)$ | (20) |
| --- | --- |

We assumed that the age distribution of the RSV-attributable ILI cases across age-groups in season $y$, $R_{y}$, is given by a multinomial distribution with probability $\sigma_{y}^{Surv}$.

| $R_{y}\sim\mathrm{Multinomial}\left( \sum_{k} R_{k,y}, \sigma_{y}^{Surv} \right)$ | (21) |
| --- | --- |

## **Virological testing**

We modelled the virological testing data using a binomial likelihood,

| $P_{j,y}\sim\mathrm{Binomial}\left( T_{j,y}, \pi_{j}^{y}\rho_{j}^{V} \right)$ | (22) |
| --- | --- |

where $T_{j,y}$ and $P_{j,y}$ are the total number of tests administered and positive test results respectively for age-group $j$ in season $y$, and $\rho_{j}^{V}$ represents the test positive probability for the RSV case selected for virological testing.

## **Likelihood formulation**

The likelihood of the model is given by the product of the likelihood defined for the hospital discharge data, RSV-attributable ILI data and virological data, i.e.

| $\mathcal{L}\left( {\sum_{k} H_{k,y},H}_{y},{\sum_{k} R_{k,y},R}_{y},P_{j,y},T_{j,y} \vert\lambda\left( a,y \right){, \rho}_{m}^{Hosp},\rho_{j}^{Surv},\rho_{j}^{V} \right)=$  $=\prod_{y} \frac{\left( \sum_{k} \Lambda_{k,y}^{Hosp} \right)^{\sum_{k} H_{k,y}}e^{\sum_{k} \Lambda_{k,y}^{Hosp}}}{\left( \sum_{k} H_{k,y} \right)!}\times\prod_{y: y\neq2020-2021} \frac{\left( \sum_{k} H_{k,y} \right)!}{\prod_{k} H_{k,y}!}\prod_{k} \theta_{k,y}^{Hosp}\times\prod_{y} \frac{\left( \sum_{k} \Lambda_{k,y}^{Surv} \right)^{\sum_{k} R_{k,y}}e^{\sum_{k} \Lambda_{k,y}^{Surv}}}{\left( \sum_{k} R_{k,y} \right)!}\times\prod_{y: y\neq2020-2021} \frac{\left( \sum_{k} R_{k,y} \right)!}{\prod_{k} R_{k,y}!}\prod_{k} \sigma_{k,y}^{Surv}\times\prod_{y} \prod_{j} \binom{T_{j,y}}{P_{j,y}}\left( \pi_{j}^{y}\rho_{j}^{V} \right)^{P_{j,y}}\left( 1-\pi_{j}^{y}\rho_{j}^{V} \right)^{T_{j,y}-P_{j,y}}$ | (23) |
| --- | --- |

We did not include the age-distribution of RSV-attributable ILI cases in 2020-2021 in the likelihood since there were zero cases reported.

## **Proportion Fully Susceptible to RSV**

To quantify the gap in immunity in the Lombardy population following the easing of COVID-19 related restrictions, we reconstructed the population immunity profile. To do this, we first compute the proportion of individuals of age $a$ in season $y$ who had previously been exposed to RSV. We retrieve this value before the start of and at the end of each season, which we call $\delta_{a,y}^{BEFORE}$ and $\delta_{a,y}^{AFTER}$. These values are given by the following equations.

| $\delta_{a,y}^{BEFORE}=\left( 1-\bar{P}_{a=0}^{\left( y-a \right),1}\left( e^{-\sum_{i=1}^{a-1} \lambda\left( i,y-a+i \right)} \right) \right)$ | (24) |
| --- | --- |

| $\delta_{a,y}^{AFTER}=\left( 1-\bar{P}_{a=0}^{\left( y-a \right),1}\left( e^{-\sum_{i=1}^{a} \lambda\left( i,y-a+i \right)} \right) \right)$ | (25) |
| --- | --- |

The overall proportions of the population naïve to RSV at the start and end of season $y$, denoted as $\mathrm{Naive}_{y}^{BEFORE}$ and $\mathrm{Naive}_{y}^{AFTER}$ respectively, are given by the following equations.

| $\mathrm{Naive}_{y}^{BEFORE}=1-\frac{\sum_{a} \delta_{a,y}^{BEFORE}\times N_{a}^{y}}{\sum_{a} N_{a}^{y}}$ | (26) |
| --- | --- |

| $\mathrm{Naive}_{y}^{AFTER}=1-\frac{\sum_{a} \delta_{a,y}^{AFTER}\times N_{a}^{y}}{\sum_{a} N_{a}^{y}}$ | (27) |
| --- | --- |

The immunity gap ($\Delta_{y}$) in any given season $y$ can be computed as the difference in proportion RSV naïve relative to the pre-pandemic season, 2018–2019.

| $\Delta_{y}=\mathrm{Naive}_{y}^{BEFORE}-\mathrm{Naive}_{2018-2019}^{BEFORE}$ | (28) |
| --- | --- |

## **Estimating Transmission Rates**

We estimate RSV transmission rates from model estimated FOI values. As a proxy for the average number of infectious individuals during some season $y$, we use the number of RSV-attributable ILI cases. The transmission rate in age-group $a$ in season $y$, $\Theta_{a,y}$, is then given by the following

| $\Theta_{a,y}=\frac{\lambda\left( a,y \right)}{\sum_{k} R_{k,y}}$ | (29) |
| --- | --- |

where $\sum_{k} R_{k,y}$ is the total number of RSV-attributable ILI cases reported in season $y$. These values $\times$100 000 are presented in the main text.

## **Immunity Profile Prior to 2022–2023**

To estimate the proportion of the population fully susceptible to RSV at the start of the 2022–2023 season, we first compute the age-stratified population in the following season, $N_{a}^{2023}$, by applying the latest 2020 mortality rate data from ISTAT^3^ and then ageing the population by one year, assuming that all individuals aged greater than 100 years are removed from the population. This is given by the following equation (for $2\leq a\leq100$).

| $N_{a}^{2023}=N_{a-1}^{2022}-\left( \frac{N_{a-1}^{2022}}{1 000}\times D_{a-1} \right)$ | (30) |
| --- | --- |

where $D_{a-1}$ is the number of deaths per 1 000 individuals age $a-1$. For the 2023 population of 1-year-olds, we combine the population estimates of those younger than one in the previous year.

| $N_{1}^{2023}=\left( N_{0-6m}^{2022}-\left( \frac{N_{0-6m}^{2022}}{1000}\times D_{0-6m} \right) \right)+\left( N_{7-12m}^{2022}-\left( \frac{N_{7-12m}^{2022}}{1000}\times D_{7-12m} \right) \right)$ | (31) |
| --- | --- |

We assume that the size of the birth cohort in the following year is the same as in 2022.

| $N_{0-6m}^{2023}=N_{0-6m}^{2022}$ | (32) |
| --- | --- |

| $N_{7-12m}^{2023}=N_{7-12m}^{2022}$ | (33) |
| --- | --- |

To compute the age-stratified number of individuals who have previously had RSV at the start of the 2022–2023 season, $I_{a}^{2023}$, we make use of the proportion of the population who have previously had RSV at the end of 2021–2022, $\delta_{a,2021-2022}^{AFTER}$, and assume that the death rate stays constant between those who have and have not had RSV before. This is given by the following equation when $2\leq a\leq100$.

| $I_{a}^{2023}=\left( \delta_{a-1, 2021-2022}^{AFTER} \right)N_{a}^{2023}$ | (34) |
| --- | --- |

Similarly, the number of 1-year-old individuals who have previously had RSV is given by combining the number previously infected among 0–6 months-old and 7–12 months-old individuals at the end of the previous season.

| $I_{1}^{2023}=\delta_{0-6m,2021-2022}^{AFTER}\left( N_{0-6m}^{2022}-\left( \frac{N_{0-6m}^{2022}}{1000}\times D_{0-6m} \right) \right)+\delta_{7-12m,2021-2022}^{AFTER}\left( N_{7-12m}^{2022}-\left( \frac{N_{7-12m}^{2022}}{1000}\times D_{7-12m} \right) \right)$ | (35) |
| --- | --- |

We assume that all individuals in the new birth cohort are RSV-naïve.

| $I_{0-6m}^{2023}=I_{7-12m}^{2023}=0$ | (36) |
| --- | --- |

The proportion of the population naïve to RSV at the start of the 2022–2023 season, $\mathrm{Naive}_{2022-2023}^{BEFORE}$, is then given by the following equation.

| $\mathrm{Naive}_{2022-2023}^{BEFORE}=1-\frac{\sum_{a} I_{a}^{2023}}{\sum_{a} N_{a}^{2023}}$ | (37) |
| --- | --- |

## **Model Parameters**

**Table S1.Prior distributions and parameter values used for model calibration.**

| **Parameter** | **Prior/Value** | **Description** |
| --- | --- | --- |
| $\lambda_{y}$ | $\lambda_{2020-2021}\sim Exp(1000)$  $\lambda_{y}\sim N(0.673977, 0.2)$ | The number of $\lambda$ values estimated depends on the model variant. For Model A, we estimated a single parameter for all age-groups, but for models B and C, we calibrate $\lambda_{y}^{C}$ and $\lambda_{y}^{A}$. In Model D, we calibrate $\lambda_{y}^{C}$, $\lambda_{y}^{Y}$, and $\lambda_{y}^{A}$ separately. Regardless of the number of $\lambda$ values estimated, we used the same prior distribution. The mean of the normal distribution is an average of the values reported in the literature.^4–9^ A special prior is set for the 2020–2021 season since the FOI was close to 0. |
| $\rho_{m}^{Hosp}$ | $\rho_{m}^{Hosp}\sim N\left( p_{m}^{Hosp},0.2 \right)$ | Mean value used in the normal distributions are based on the mean estimates from van Boven *et al*. calibrating a model of RSV transmission in the Netherlands.^10^ The variables $p^{Hosp}$ and $p^{GP}$ correspond to the probabilities of hospitalisation and general practitioner (GP) consultation respectively.^10^ |
| $\rho_{j}^{Surv}$ | $\rho_{j}^{Surv}\sim N\left( p_{j}^{GP},0.2 \right)$ |  |
| $\rho_{j}^{V}$ | $\rho_{j}^{V}\sim N\left( p_{j}^{GP},0.2 \right)$ |  |
| $s$ | Fixed $s=0.77$ | Value is based on a pooled estimate by Paynter *et al*. derived from experimental infection studies in adults.^8^ |

## **Prior distribution for** $\boldsymbol{\lambda}$

The prior distribution for $\lambda$ is given in Table S1. This prior applies to $\lambda_{y}^{C}$, $\lambda_{y}^{Y}$, and $\lambda_{y}^{A}$, depending on whether the value is calibrated according to the model variant. We found that Model D was favoured by the DIC, and in this case $\lambda_{y}^{C}$, $\lambda_{y}^{Y}$, and $\lambda_{y}^{A}$ are each calibrated separately, but they start from the same prior distribution. The mean value of this prior is based on two types of data: (1) estimates of the proportion of 1- and 2-year-olds who have experienced primary RSV infection, and (2) direct estimates of the RSV FOI.

Assuming a constant RSV FOI $\lambda$, the probability an individual of age $a$ has been infected by RSV at least once before, which we call $W_{a}$, is given by the following equation.

| $W_{a}=1-e^{-a\lambda}$ | (38) |
| --- | --- |

Note that this assumes an individual age $a$ experiences only $a$ RSV seasons. For example, a 2-year-old experiences 2 RSV seasons: one as a 0-year-old, and one as a 1-year-old. What we generally know from studies is the value of $W_{a}$. We can solve for $\lambda$ to estimate the constant average FOI from the value of $W_{a}$. This results in the following equation for $\lambda$.

| $\lambda=-\frac{\log\left( 1-W_{a} \right)}{a}$ | (39) |
| --- | --- |

For each data source, we use this equation to compute an estimate for $\lambda$. The value for 1-year-olds and 2-year-olds is then averaged to get an overall average value. These values are all summarised in Table S2 below.

**Table S2. Summary of estimated** $\boldsymbol{\lambda}$ **values given** $\boldsymbol{W}_{\boldsymbol{a}}\boldsymbol{.}$

| **Source** | $\boldsymbol{W}_{\boldsymbol{a}}$ | $\lambda$ **estimate** | **Average** $\lambda$ |
| --- | --- | --- | --- |
| Public Health England^9^ | $W_{1}=0.6$ | $0.9163$ | $0.860505$ |
|  | $W_{2}=0.8$ | $0.8047$ |  |
| Andeweg *et al*.^4^ | $W_{1}=0.441$ | $0.5816$ | $0.758504$ |
|  | $W_{2}=0.846$ | $0.9354$ |  |
| Kutsaya *et al.*^6^ | $W_{1}=0.37$ | $0.4620$ | $0.515876$ |
|  | $W_{2}=0.68$ | $0.5697$ |  |

We take these average $\lambda$ values then combine them with some direct estimates of the RSV FOI. From a study of 635 children in Kilifi, Kenya, Ohuma *et al*. report an estimated incidence rate of 495 cases per 1 000 child-years (which we translate to an FOI of 0.495).^7^ A study of a birth cohort of 125 children found in Houston, USA, found the FOI to be around 0.74 (study by Glezen *et al.* but we use the pooled value presented by Paynter *et al.*).^5,8^ We summarise the values we have collected in Table S3 below.

**Table S3. Summary of FOI estimates used as mean of the prior distributions.** Values marked with a * are computed values as seen in Table S2.

| **Source** | **Estimate of** $\lambda$ |
| --- | --- |
| Public Health England^9^* | 0.860505 |
| Andeweg *et al.*^4^* | 0.758504 |
| Kutsaya *et al.*^6^* | 0.515876 |
| Ohuma *et al.*^7^ | 0.495 |
| Glezen *et al.* (via Paynter *et al.*)^5,8^ | 0.74 |

Taking the mean value of all these $\lambda$ estimates, we have the value used as the mean for the prior of $\lambda$, 0.673977. We choose a standard deviation of 0.2 since that value around the mean roughly contains all the $\lambda$ estimates collected here.

## **Prior distribution for** $\boldsymbol{\rho}^{\boldsymbol{Hosp}}$**,** $\boldsymbol{\rho}^{\boldsymbol{Surv}}$**,** $\boldsymbol{\rho}^{\boldsymbol{V}}$

Table S4 summarises the prior distributions used for the model parameters $\rho_{m}^{Hosp}$, $\rho_{j}^{Surv}$, and $\rho_{j}^{V}$, which were informed by the estimates in van Boven *et al*..^10^

**Table S4. Model parameters and values used for the mean of the prior distributions.** Values from van Boven *et al.*^10^, Normal prior distributions used with standard deviation $\sigma=0.2$. Subscripts denote age-groups.

| **Model Parameter** | **Prior distribution Mean** |
| --- | --- |
| $\rho_{0-6 \mathrm{months}}^{Hosp}$ | $p_{0}^{Hosp}= 0.014$ |
| $\rho_{7-12 \mathrm{months}}^{Hosp}$ | $p_{0}^{Hosp}= 0.014$ |
| $\rho_{1}^{Hosp}$ | $p_{1-4}^{Hosp}= 0.0015$ |
| $\rho_{2}^{Hosp}$ | $p_{1-4}^{Hosp}= 0.0015$ |
| $\rho_{3+}^{Hosp}$ | $p_{5-64}^{Hosp}= 0.00070$ |
| $\rho_{0-4}^{Surv}$, $\rho_{0-4}^{V}$ | $p_{1-4}^{GP}=0.19$ |
| $\rho_{5-14}^{Surv}$, $\rho_{5-14}^{V}$ | $p_{5-64}^{GP}=0.17$ |
| $\rho_{15-64}^{Surv}$, $\rho_{15-64}^{V}$ | $p_{5-64}^{GP}=0.17$ |
| $\rho_{65+}^{Surv}$, $\rho_{65+}^{V}$ | $p_{65+}^{GP}=0.15$ |

## **Model variables**

**Table S5. Model variables, data types, values, and descriptions.**

| **Variable** | **Type** | **Value** | **Description** |
| --- | --- | --- | --- |
| $\lambda_{y}^{C}$ | Continuous | Fitted | FOI among individuals aged 0–4 years in season $y$ |
| $\lambda_{y}^{Y}$ | Continuous | Fitted | FOI among individuals aged 5–14 years in season $y$ |
| $\lambda_{y}^{A}$ | Continuous | Fitted | FOI among individuals aged 15+ years in season $y$ |
| $\lambda(a,y)$ | Continuous | Function | FOI of an individual aged $a$ in season $y$ depending on the values of $\lambda_{y}^{C}$, $\lambda_{y}^{Y}$, and $\lambda_{y}^{A}$. |
| $s$ | Continuous | Fixed | Factor denoting the reduced susceptibility to post-primary infection |
| $P_{a}^{y,1}$ | Continuous | Computed | Probability of primary infection for an individual aged $a$ in season $y$ |
| $P_{a}^{y,2}$ | Continuous | Computed | Probability of post-primary infection for an individual aged $a$ in season $y$ |
| $\bar{P}_{0}^{y,1}$ | Continuous | Computed | Probability of individual escaping primary infection in their first year of life in season $y$ |
| $P_{a}^{y}$ | Continuous | Computed | Probability of RSV infection for an individual aged $a$ in season $y$ |
| $\pi_{g}^{y}$ | Continuous | Computed | Probability of infection for an individual in age-group $g$ in season $y$ |
| $H_{g,w}$ | Count | Data | Number of hospital discharges in age-group $g$ in week $w$ |
| $H_{g,y}$ | Count | Data | Number of hospital discharges in age-group $g$ in season $y$ (computed from weekly values) |
| $\rho_{m}^{Hosp}$ | Continuous | Fitted | Probability of hospitalisation for individuals in age-group $m$ |
| $\Lambda_{g,y}^{Hosp}$ | Continuous | Computed | Model computed expected number of hospitalisations in age-group $g$ in season $y$ |
| $\theta_{g,y}^{Hosp}$ | Continuous | Computed | Model computed proportion of hospitalisations in season $y$ that are in age-group $g$ |
| $I_{j,w}$ | Continuous | Data | ILI incidence per 1000 individuals in age-group $j$ in week $w$ |
| $P_{j,w}$ | Count | Data | Number of positive RSV tests in age-group $j$ in week $w$ |
| $P_{j,y}$ | Count | Data | Number of positive RSV tests in age-group $j$ in season $y$ |
| $T_{j,w}$ | Count | Data | Number of RSV tests carried out in age-group $j$ in week $w$ |
| $T_{j,y}$ | Count | Data | Number of RSV tests carried out in age-group $j$ in season $y$ |
| $N_{j,y}$ | Count | Data | Population of age-group $j$ in season $y$ |
| $R_{j,y}$ | Count | Computed | Number of RSV-attributable ILI cases in age-group $j$ in season $y$ |
| $\rho_{j}^{Surv}$ | Continuous | Fitted | Probability that an RSV infection in age-group $j$ is symptomatic and reported to surveillance |
| $\Lambda_{j,y}^{Surv}$ | Continuous | Computed | Model computed expected number of RSV-attributable ILI cases in age-group $j$ in season $y$ |
| $\sigma_{j,y}^{Surv}$ | Continuous | Computed | Model computed proportion of RSV-attributable ILI cases in age-group $j$ in season $y$ |
| $\rho_{j}^{V}$ | Continuous | Fitted | Test positive probability in age-group $j$ |
| $\delta_{a,y}^{BEFORE}$ | Continuous | Computed | Proportion of individuals aged $a$ who have previously had RSV at the start of season $y$ |
| $\delta_{a,y}^{AFTER}$ | Continuous | Computed | Proportion of individuals aged $a$ who have previously had RSV at the end of season $y$ |
| $\mathrm{Naive}_{y}^{BEFORE}$ | Continuous | Computed | Proportion of the Lombardy population at the start of season $y$ who are naïve to RSV |
| $\mathrm{Naive}_{y}^{AFTER}$ | Continuous | Computed | Proportion of the Lombardy population at the end of season $y$ who are naïve to RSV |
| $\Delta_{y}$ | Continuous | Computed | Immunity gap in season $y$ relative to the pre-pandemic season 2018–2019. |
| $\Theta_{a,y}$ | Continuous | Computed | Estimated transmission rate in age-group $a$ in season $y$, computed from fitted FOI values and the number of RSV-attributable ILI cases from data |
| $D_{a}$ | Continuous | Data | Death rate per 1 000 individuals age $a$ for the year 2020 |
| $I_{a}^{2023}$ | Continuous | Computed | Proportion of individuals aged $a$ who have previously had RSV at the start of the 2022–2023 season |
| $r$ | Continuous | Fitted | Parameter denoting reduced probability of hospitalisation for those with post-primary RSV infection (used only in sensitivity analysis). |

## **Model selection**

We used the Deviance Information Criterion (DIC) for model selection, which is given by^11^

| $\mathrm{DIC}=-2\log\left( p\left( d \vert\hat{\theta} \right) \right)+2p\mathrm{DIC}$ | (40) |
| --- | --- |

The effective number of parameters $\mathrm{pDIC}$ is given by the following equation^11^

| $\mathrm{pDIC}=2\left( \log\left( p\left( d \vert\hat{\theta} \right) \right)-E_{post}\left( \log\left( p\left( d \vert\theta\right) \right) \right) \right)$ | (41) |
| --- | --- |

where $d$ is the data we are fitting to, $\hat{\theta}$ is the posterior mean, $\log\left( p\left( d | \hat{\theta} \right) \right)$ is the log-likelihood evaluated at the posterior mean, and $E_{post}\left( \log\left( p(d|\theta) \right) \right)$ is the mean of the posterior log-likelihood.

Smaller DIC values indicate preferred models, having accounted for model complexity.

## **Sensitivity Analysis: Increased probability of reporting to surveillance (**$\boldsymbol{\rho}^{\boldsymbol{Surv}}$**) during seasons affected by the pandemic**

We explored scenarios where the probability of RSV infections being symptomatic and reported to surveillance is increased by 25% and 50% during seasons affected by the COVID-19 pandemic (seasons from 2019 to 2022). More specifically, the equation for the expected number of RSV-attributable ILI cases in age-group $j$ during season $y$ ($\Lambda_{j,y}^{Surv}$) is given as the following

| $\Lambda_{j,y}^{Surv}=\pi_{j}^{y}\rho_{j}^{Surv}qN_{j}^{y}$ | (42) |
| --- | --- |

where $q=1.25$ or $q=1.5$ (depending on the scenario) when $y\in\{2019-2020, 2020-2021, 2021-2022\}$ and $q=1$ otherwise.

## **Sensitivity Analysis: Calibrated factor** $\boldsymbol{r}$ **for reduced probability of hospitalisation for post-primary RSV infections**

The relationship between prior exposure to RSV and the pathogenesis of severe disease is often complicated by age as a confounding factor since those experiencing post-primary infection are generally older.^7^ In this sensitivity analysis, we introduce a new calibrated parameter, $r\in[0,1]$, a factor denoting a reduced probability of hospitalisation for post-primary RSV infections. We refer to this model as “calibrated $r$.” Let $\pi_{g}^{y,1}$ and $\pi_{g}^{y,2}$ be the probabilities of primary and post-primary RSV infection for an individual in age-group $g$ in season $y$. The expected number of hospitalisations in age-group $g$ in season $y$, $\Lambda_{g,y}^{Hosp}$, is then given by the following equation.

| $\Lambda_{g,y}^{Hosp}=\left( \pi_{g}^{y,1}\rho_{m}^{Hosp}N_{g}^{y} \right)+\left( \pi_{g}^{y,2}r\rho_{m}^{Hosp}N_{g}^{y} \right)$ | (43) |
| --- | --- |

We fit the model assuming a $U(0,1)$ prior for $r$.

# **Results**

## **Parameter estimates and model fit – scenarios with MDI (**$\boldsymbol{\mu}$ **= 1)**

**Table S6. Mean and 95% CrI of estimated parameters and main results of models with MDI (**$\boldsymbol{\mu=1}$**).** We used NA when the parameter was not used for the given model variant. For example, values of $\lambda^{C}$ are given as NA under Model A since it is set to the value of $\lambda^{A}$. The % change in proportion naïve refers to the proportion RSV naïve in 2021–2022 versus 2018–2019.

| Parameter | Model A | Model B | Model C | Model D (Baseline) |
| --- | --- | --- | --- | --- |
| $\boldsymbol{\lambda}_{\boldsymbol{2018-2019}}^{\boldsymbol{A}}$ | 1.619 (1.359–1.914) | 0.366 (0.293–0.456) | 0.113 (0.092–0.138) | 0.113 (0.091–0.141) |
| $\boldsymbol{\lambda}_{\boldsymbol{2019-2020}}^{\boldsymbol{A}}$ | 0.388 (0.350–0.426) | 0.099 (0.081–0.121) | 0.030 (0.024–0.036) | 0.033 (0.027–0.041) |
| $\boldsymbol{\lambda}_{\boldsymbol{2020-2021}}^{\boldsymbol{A}}$ | 0.000 (0.000–0.000) | 0.000 (0.000–0.000) | 0.000 (0.000–0.000) | 0.000 (0.000–0.000) |
| $\boldsymbol{\lambda}_{\boldsymbol{2021-2022}}^{\boldsymbol{A}}$ | 1.075 (0.934–1.223) | 0.251 (0.203–0.310) | 0.076 (0.062–0.093) | 0.080 (0.064–0.099) |
| $\boldsymbol{\lambda}_{\boldsymbol{2018-2019}}^{\boldsymbol{C}}$ | NA | 1.189 (1.026–1.368) | 1.136 (0.982–1.296) | 1.126 (0.962–1.287) |
| $\boldsymbol{\lambda}_{\boldsymbol{2019-2020}}^{\boldsymbol{C}}$ | NA | 0.423 (0.380–0.467) | 0.588 (0.524–0.652) | 0.584 (0.515–0.649) |
| $\boldsymbol{\lambda}_{\boldsymbol{2020-2021}}^{\boldsymbol{C}}$ | NA | 0.001 (0.000–0.001) | 0.001 (0.001–0.001) | 0.001 (0.001–0.001) |
| $\boldsymbol{\lambda}_{\boldsymbol{2021-2022}}^{\boldsymbol{C}}$ | NA | 1.036 (0.900–1.183) | 1.421 (1.196–1.666) | 1.406 (1.171–1.653) |
| $\boldsymbol{\lambda}_{\boldsymbol{2018-2019}}^{\boldsymbol{Y}}$ | NA | NA | NA | 0.187 (0.135–0.252) |
| $\boldsymbol{\lambda}_{\boldsymbol{2019-2020}}^{\boldsymbol{Y}}$ | NA | NA | NA | 0.025 (0.018–0.033) |
| $\boldsymbol{\lambda}_{\boldsymbol{2020-2021}}^{\boldsymbol{Y}}$ | NA | NA | NA | 0.000 (0.000–0.000) |
| $\boldsymbol{\lambda}_{\boldsymbol{2021-2022}}^{\boldsymbol{Y}}$ | NA | NA | NA | 0.095 (0.069–0.126) |
| $\boldsymbol{\rho}_{\boldsymbol{0-6}\mathbf{mos.}}^{\boldsymbol{Hosp}}$ | 0.238 (0.221–0.258) | 0.253 (0.233–0.275) | 0.227 (0.210–0.247) | 0.228 (0.211–0.250) |
| $\boldsymbol{\rho}_{\boldsymbol{7-12}\mathbf{mos.}}^{\boldsymbol{Hosp}}$ | 0.010 (0.009–0.011) | 0.010 (0.009–0.011) | 0.009 (0.008–0.010) | 0.009 (0.008–0.010) |
| $\boldsymbol{\rho}_{\boldsymbol{1}}^{\boldsymbol{Hosp}}$ | 0.004 (0.004–0.004) | 0.004 (0.004–0.005) | 0.004 (0.003–0.004) | 0.004 (0.003–0.004) |
| $\boldsymbol{\rho}_{\boldsymbol{2}}^{\boldsymbol{Hosp}}$ | 0.002 (0.001–0.002) | 0.002 (0.001–0.002) | 0.002 (0.001–0.002) | 0.002 (0.001–0.002) |
| $\boldsymbol{\rho}_{\boldsymbol{3+}}^{\boldsymbol{Hosp}}$ | 0.000 (0.000–0.000) | 0.000 (0.000–0.000) | 0.000 (0.000–0.000) | 0.000 (0.000–0.000) |
| $\boldsymbol{\rho}_{\boldsymbol{0-4}}^{\boldsymbol{Surv}}$ | 0.231 (0.214–0.251) | 0.246 (0.228–0.267) | 0.220 (0.204–0.239) | 0.221 (0.205–0.242) |
| $\boldsymbol{\rho}_{\boldsymbol{5-14}}^{\boldsymbol{Surv}}$ | 0.029 (0.027–0.032) | 0.031 (0.029–0.034) | 0.274 (0.223–0.330) | 0.202 (0.150–0.267) |
| $\boldsymbol{\rho}_{\boldsymbol{15-64}}^{\boldsymbol{Surv}}$ | 0.019 (0.017–0.020) | 0.058 (0.048–0.070) | 0.177 (0.144–0.213) | 0.171 (0.138–0.210) |
| $\boldsymbol{\rho}_{\boldsymbol{65+}}^{\boldsymbol{Surv}}$ | 0.017 (0.015–0.018) | 0.052 (0.043–0.062) | 0.158 (0.128–0.190) | 0.153 (0.123–0.187) |
| $\boldsymbol{\rho}_{\boldsymbol{0-4}}^{\boldsymbol{V}}$ | 0.626 (0.547–0.711) | 0.645 (0.563–0.734) | 0.555 (0.484–0.631) | 0.557 (0.486–0.636) |
| $\boldsymbol{\rho}_{\boldsymbol{5-14}}^{\boldsymbol{V}}$ | 0.164 (0.108–0.230) | 0.168 (0.110–0.237) | 0.788 (0.569–0.979) | 0.745 (0.521–0.962) |
| $\boldsymbol{\rho}_{\boldsymbol{15-64}}^{\boldsymbol{V}}$ | 0.114 (0.083–0.149) | 0.353 (0.251–0.476) | 0.805 (0.612–0.979) | 0.787 (0.591–0.971) |
| $\boldsymbol{\rho}_{\boldsymbol{65+}}^{\boldsymbol{V}}$ | 0.239 (0.144–0.352) | 0.547 (0.346–0.781) | 0.749 (0.510–0.969) | 0.744 (0.503–0.965) |
| $\boldsymbol{\Delta}_{\boldsymbol{2018-2019}}^{\boldsymbol{BEFORE}}$ | 0.012 (0.012–0.013) | 0.014 (0.013–0.015) | 0.015 (0.013–0.016) | 0.014 (0.013–0.016) |
| $\boldsymbol{\Delta}_{\boldsymbol{2021-2022}}^{\boldsymbol{BEFORE}}$ | 0.023 (0.022–0.024) | 0.024 (0.023–0.025) | 0.023 (0.022–0.025) | 0.023 (0.022–0.025) |
| % Change Proportion Naive | 88.1% (83.0–92.8%) | 72.5% (67.7–77.1%) | 60.3% (54.7–65.2%) | 60.8% (55.2–65.4%) |
| Immunity Gap $\boldsymbol{(}\boldsymbol{\Delta}_{\boldsymbol{2021-2022}}\boldsymbol{)}$ | 1.072% (1.068–1.075%) | 0.995% (0.990–0.999%) | 0.873% (0.867–0.880%) | 0.874% (0.867–0.880%) |
| % Change Proportion Naive  (1–5 years) | 272.1% (233.4–311.9%) | 191.5% (165.5–219.2%) | 162.8% (140.6–185.1%) | 161.3% (137.7–183.9%) |
| Immunity Gap ($\boldsymbol{\Delta}_{\boldsymbol{2021-2022}}\boldsymbol{)}$  1–5 years | 29.987% (29.837–30.114%) | 28.102% (27.983–28.221%) | 24.968% (24.799–25.135%) | 24.966% (24.792–25.136%) |

## **Sensitivity Analysis: Scenarios without MDI (**$\boldsymbol{\mu}$ **= 0)**

**Table S7. Mean and 95% CrI of estimated parameters and main results of models without MDI (**$\boldsymbol{\mu=0}$**).** We use NA when the parameter was not used for the given model variant. For example, values of $\lambda^{C}$ are given as NA under Model A since it is set to the value of $\lambda^{A}$. The % change in proportion naïve refers to the proportion RSV naïve in 2021-2022 versus 2018-2019.

| Parameter | Model A | Model B | Model C | Model D |
| --- | --- | --- | --- | --- |
| $\boldsymbol{\lambda}_{\boldsymbol{2018-2019}}^{\boldsymbol{A}}$ | 1.431 (1.197–1.709) | 0.350 (0.279–0.438) | 0.112 (0.091–0.137) | 0.113 (0.090–0.140) |
| $\boldsymbol{\lambda}_{\boldsymbol{2019-2020}}^{\boldsymbol{A}}$ | 0.361 (0.322–0.402) | 0.095 (0.077–0.116) | 0.030 (0.024–0.036) | 0.033 (0.026–0.040) |
| $\boldsymbol{\lambda}_{\boldsymbol{2020-2021}}^{\boldsymbol{A}}$ | 0.000 (0.000–0.000) | 0.000 (0.000–0.000) | 0.000 (0.000–0.000) | 0.000 (0.000–0.000) |
| $\boldsymbol{\lambda}_{\boldsymbol{2021-2022}}^{\boldsymbol{A}}$ | 0.976 (0.841–1.125) | 0.241 (0.194–0.298) | 0.075 (0.061–0.092) | 0.080 (0.064–0.099) |
| $\boldsymbol{\lambda}_{\boldsymbol{2018-2019}}^{\boldsymbol{C}}$ | NA | 1.113 (0.952–1.293) | 1.120 (0.962–1.281) | 1.119 (0.956–1.282) |
| $\boldsymbol{\lambda}_{\boldsymbol{2019-2020}}^{\boldsymbol{C}}$ | NA | 0.404 (0.359–0.450) | 0.583 (0.516–0.647) | 0.583 (0.514–0.647) |
| $\boldsymbol{\lambda}_{\boldsymbol{2020-2021}}^{\boldsymbol{C}}$ | NA | 0.001 (0.000–0.001) | 0.001 (0.001–0.001) | 0.001 (0.001–0.001) |
| $\boldsymbol{\lambda}_{\boldsymbol{2021-2022}}^{\boldsymbol{C}}$ | NA | 0.976 (0.840–1.127) | 1.400 (1.172–1.644) | 1.399 (1.163–1.648) |
| $\boldsymbol{\lambda}_{\boldsymbol{2018-2019}}^{\boldsymbol{Y}}$ | NA | NA | NA | 0.186 (0.134–0.252) |
| $\boldsymbol{\lambda}_{\boldsymbol{2019-2020}}^{\boldsymbol{Y}}$ | NA | NA | NA | 0.025 (0.018–0.033) |
| $\boldsymbol{\lambda}_{\boldsymbol{2020-2021}}^{\boldsymbol{Y}}$ | NA | NA | NA | 0.000 (0.000–0.000) |
| $\boldsymbol{\lambda}_{\boldsymbol{2021-2022}}^{\boldsymbol{Y}}$ | NA | NA | NA | 0.094 (0.069–0.126) |
| $\boldsymbol{\rho}_{\boldsymbol{0-6}\mathbf{mos.}}^{\boldsymbol{Hosp}}$ | 0.084 (0.077–0.092) | 0.087 (0.080–0.096) | 0.076 (0.070–0.083) | 0.076 (0.070–0.084) |
| $\boldsymbol{\rho}_{\boldsymbol{7-12}\mathbf{mos.}}^{\boldsymbol{Hosp}}$ | 0.010 (0.009–0.012) | 0.011 (0.009–0.012) | 0.009 (0.008–0.010) | 0.009 (0.008–0.010) |
| $\boldsymbol{\rho}_{\boldsymbol{1}}^{\boldsymbol{Hosp}}$ | 0.004 (0.004–0.005) | 0.004 (0.004–0.005) | 0.004 (0.003–0.004) | 0.004 (0.003–0.004) |
| $\boldsymbol{\rho}_{\boldsymbol{2}}^{\boldsymbol{Hosp}}$ | 0.002 (0.001–0.002) | 0.002 (0.002–0.002) | 0.002 (0.001–0.002) | 0.002 (0.001–0.002) |
| $\boldsymbol{\rho}_{\boldsymbol{3+}}^{\boldsymbol{Hosp}}$ | 0.000 (0.000–0.000) | 0.000 (0.000–0.000) | 0.000 (0.000–0.000) | 0.000 (0.000–0.000) |
| $\boldsymbol{\rho}_{\boldsymbol{0-4}}^{\boldsymbol{Surv}}$ | 0.231 (0.212–0.253) | 0.241 (0.221–0.263) | 0.209 (0.194–0.228) | 0.209 (0.193–0.229) |
| $\boldsymbol{\rho}_{\boldsymbol{5-14}}^{\boldsymbol{Surv}}$ | 0.031 (0.028–0.034) | 0.032 (0.029–0.036) | 0.276 (0.225–0.334) | 0.203 (0.150–0.270) |
| $\boldsymbol{\rho}_{\boldsymbol{15-64}}^{\boldsymbol{Surv}}$ | 0.020 (0.018–0.022) | 0.060 (0.049–0.073) | 0.178 (0.145–0.216) | 0.172 (0.138–0.211) |
| $\boldsymbol{\rho}_{\boldsymbol{65+}}^{\boldsymbol{Surv}}$ | 0.018 (0.016–0.020) | 0.054 (0.044–0.065) | 0.159 (0.130–0.192) | 0.153 (0.123–0.188) |
| $\boldsymbol{\rho}_{\boldsymbol{0-4}}^{\boldsymbol{V}}$ | 0.626 (0.544–0.715) | 0.631 (0.549–0.720) | 0.527 (0.459–0.603) | 0.528 (0.459–0.604) |
| $\boldsymbol{\rho}_{\boldsymbol{5-14}}^{\boldsymbol{V}}$ | 0.174 (0.114–0.247) | 0.174 (0.113–0.247) | 0.788 (0.569–0.980) | 0.747 (0.522–0.964) |
| $\boldsymbol{\rho}_{\boldsymbol{15-64}}^{\boldsymbol{V}}$ | 0.121 (0.088–0.160) | 0.365 (0.259–0.492) | 0.808 (0.615–0.978) | 0.788 (0.594–0.971) |
| $\boldsymbol{\rho}_{\boldsymbol{65+}}^{\boldsymbol{V}}$ | 0.253 (0.154–0.372) | 0.559 (0.353–0.797) | 0.750 (0.510–0.969) | 0.744 (0.504–0.968) |
| $\boldsymbol{\Delta}_{\boldsymbol{2018-2019}}^{\boldsymbol{BEFORE}}$ | 0.010 (0.009–0.011) | 0.011 (0.011–0.013) | 0.012 (0.011–0.013) | 0.012 (0.011–0.013) |
| $\boldsymbol{\Delta}_{\boldsymbol{2021-2022}}^{\boldsymbol{BEFORE}}$ | 0.021 (0.020–0.022) | 0.022 (0.021–0.023) | 0.021 (0.020–0.022) | 0.020 (0.019–0.022) |
| % Change Proportion Naïve | 107.6% (98.6–116.5%) | 87.6% (79.7–95.3%) | 75.3% (67.0–82.5%) | 76.0% (68.0–82.9%) |
| Immunity Gap  $\boldsymbol{(}\boldsymbol{\Delta}_{\boldsymbol{2021-2022}}\boldsymbol{)}$ | 1.081% (1.078–1.084%) | 1.003% (0.999–1.008%) | 0.883% (0.876–0.889%) | 0.883% (0.876–0.889%) |
| % Change Proportion Naïve (1–5 years) | 505.4% (363.4–708.1%) | 301.9% (233.7–387.9%) | 270.8% (211.1–338.8%) | 270.5% (209.3–339.3%) |
| Immunity Gap ($\boldsymbol{\Delta}_{\boldsymbol{2021-2022}}\boldsymbol{)}$  1–5 years | 29.795% (29.631–29.927%) | 27.916% (27.792–28.034%) | 24.806% (24.637–24.974%) | 24.805% (24.632–24.973%) |

## **Sensitivity Analysis: Increased probability of reporting to surveillance (**$\boldsymbol{\rho}^{\boldsymbol{Surv}}$**) during seasons affected by the pandemic**

Model fit versus baseline is visualised in Figure S4, while parameter estimates of this analysis are summarised in Table S8.

The proportion of individuals naïve to RSV increases from 1.2% (95% CrI: 1.2–1.3%) in 2018–2019 to 2.2% (95% CrI: 2.1–2.3%) in 2021–2022 with a 25% increase in reporting, while the increase is from 1.2% (95% CrI: 1.1–1.2%) to 2.2% (95% CrI: 2.1–2.2%) with a 50% increase in reporting. These results correspond to an immunity gap ($\Delta_{2021-2022})$ of 0.96% (95% CrI: 0.96–0.97%) and 1.02% (95% CrI: 1.02–1.03%), respectively. These values are slightly larger than though similar to the immunity gap at baseline (0.87% [95% CrI: 0.87–0.88%]).

**Table S8. Mean and 95% CrI of estimated parameters obtained with the baseline model and sensitivity analysis modifying the probability of RSV infections being symptomatic and reported to surveillance during the pandemic seasons.** The % change in proportion naïve refers to the proportion RSV naïve in 2021-2022 versus 2018-2019.

| Parameter | Baseline | 25% increased reporting | 50% increased reporting |
| --- | --- | --- | --- |
| $\boldsymbol{\lambda}_{\mathbf{2018-2019}}^{\boldsymbol{A}}$ | 0.113 (0.091–0.141) | 0.134 (0.108–0.166) | 0.148 (0.118–0.184) |
| $\boldsymbol{\lambda}_{\mathbf{2019-2020}}^{\boldsymbol{A}}$ | 0.033 (0.027–0.041) | 0.031 (0.025–0.038) | 0.028 (0.023–0.035) |
| $\boldsymbol{\lambda}_{\mathbf{2020-2021}}^{\boldsymbol{A}}$ | 0.000 (0.000–0.000) | 0.000 (0.000–0.000) | 0.000 (0.000–0.000) |
| $\boldsymbol{\lambda}_{\mathbf{2021-2022}}^{\boldsymbol{A}}$ | 0.080 (0.064–0.099) | 0.075 (0.061–0.093) | 0.069 (0.055–0.085) |
| $\boldsymbol{\lambda}_{\mathbf{2018-2019}}^{\boldsymbol{C}}$ | 1.126 (0.962–1.287) | 1.557 (1.328–1.798) | 1.924 (1.657–2.204) |
| $\boldsymbol{\lambda}_{\mathbf{2019-2020}}^{\boldsymbol{C}}$ | 0.584 (0.515–0.649) | 0.559 (0.503–0.611) | 0.507 (0.468–0.544) |
| $\boldsymbol{\lambda}_{\mathbf{2020-2021}}^{\boldsymbol{C}}$ | 0.001 (0.001–0.001) | 0.001 (0.001–0.001) | 0.001 (0.001–0.001) |
| $\boldsymbol{\lambda}_{\mathbf{2021-2022}}^{\boldsymbol{C}}$ | 1.406 (1.171–1.653) | 1.296 (1.117–1.479) | 1.118 (1.002–1.230) |
| $\boldsymbol{\lambda}_{\mathbf{2018-2019}}^{\boldsymbol{Y}}$ | 0.187 (0.135–0.252) | 0.217 (0.157–0.294) | 0.236 (0.170–0.321) |
| $\boldsymbol{\lambda}_{\mathbf{2019-2020}}^{\boldsymbol{Y}}$ | 0.025 (0.018–0.033) | 0.023 (0.017–0.030) | 0.020 (0.015–0.027) |
| $\boldsymbol{\lambda}_{\mathbf{2020-2021}}^{\boldsymbol{Y}}$ | 0.000 (0.000–0.000) | 0.000 (0.000–0.000) | 0.000 (0.000–0.000) |
| $\boldsymbol{\lambda}_{\mathbf{2021-2022}}^{\boldsymbol{Y}}$ | 0.095 (0.069–0.126) | 0.087 (0.064–0.116) | 0.078 (0.057–0.104) |
| $\boldsymbol{\rho}_{\mathbf{0-6 mos.}}^{\boldsymbol{Hosp}}$ | 0.228 (0.211–0.250) | 0.219 (0.204–0.236) | 0.220 (0.208–0.234) |
| $\boldsymbol{\rho}_{\mathbf{7-12 mos.}}^{\boldsymbol{Hosp}}$ | 0.009 (0.008–0.010) | 0.009 (0.008–0.010) | 0.009 (0.008–0.010) |
| $\boldsymbol{\rho}_{\mathbf{1}}^{\boldsymbol{Hosp}}$ | 0.004 (0.003–0.004) | 0.004 (0.003–0.004) | 0.004 (0.003–0.004) |
| $\boldsymbol{\rho}_{\mathbf{2}}^{\boldsymbol{Hosp}}$ | 0.002 (0.001–0.002) | 0.001 (0.001–0.002) | 0.001 (0.001–0.002) |
| $\boldsymbol{\rho}_{\mathbf{3+}}^{\boldsymbol{Hosp}}$ | 0.000 (0.000–0.000) | 0.000 (0.000–0.000) | 0.000 (0.000–0.000) |
| $\boldsymbol{\rho}_{\mathbf{0-4}}^{\boldsymbol{Surv}}$ | 0.221 (0.205–0.242) | 0.186 (0.173–0.200) | 0.168 (0.159–0.179) |
| $\boldsymbol{\rho}_{\mathbf{5-14}}^{\boldsymbol{Surv}}$ | 0.202 (0.150–0.267) | 0.176 (0.130–0.233) | 0.163 (0.121–0.215) |
| $\boldsymbol{\rho}_{\mathbf{15-64}}^{\boldsymbol{Surv}}$ | 0.171 (0.138–0.210) | 0.146 (0.118–0.178) | 0.133 (0.107–0.162) |
| $\boldsymbol{\rho}_{\mathbf{65+}}^{\boldsymbol{Surv}}$ | 0.153 (0.123–0.187) | 0.130 (0.105–0.158) | 0.118 (0.095–0.144) |
| $\boldsymbol{\rho}_{\mathbf{0-4}}^{\boldsymbol{V}}$ | 0.557 (0.486–0.636) | 0.570 (0.500–0.648) | 0.603 (0.532–0.679) |
| $\boldsymbol{\rho}_{\mathbf{5-14}}^{\boldsymbol{V}}$ | 0.745 (0.521–0.962) | 0.750 (0.525–0.967) | 0.759 (0.534–0.969) |
| $\boldsymbol{\rho}_{\mathbf{15-64}}^{\boldsymbol{V}}$ | 0.787 (0.591–0.971) | 0.791 (0.596–0.973) | 0.807 (0.611–0.981) |
| $\boldsymbol{\rho}_{\mathbf{65+}}^{\boldsymbol{V}}$ | 0.744 (0.503–0.965) | 0.746 (0.503–0.969) | 0.748 (0.509–0.968) |
| $\boldsymbol{\Delta}_{\mathbf{2018-2019}}^{\boldsymbol{BEFORE}}$ | 0.014 (0.013–0.016) | 0.012 (0.012–0.013) | 0.012 (0.011–0.012) |
| $\boldsymbol{\Delta}_{\mathbf{2021-2022}}^{\boldsymbol{BEFORE}}$ | 0.023 (0.022–0.025) | 0.022 (0.021–0.023) | 0.022 (0.021–0.022) |
| % Change Proportion Naïve | 60.8% (55.2–65.4%) | 77.9% (73.2–81.9%) | 88.7% (85.0–91.8%) |
| Immunity Gap  $\boldsymbol{(}\boldsymbol{\Delta}_{\boldsymbol{2021-2022}}\boldsymbol{)}$ | 0.874% (0.867–0.880%) | 0.963% (0.957–0.969%) | 1.024% (1.019–1.029%) |
| % Change Proportion Naïve (1–5 years) | 161.3% (137.7–183.9%) | 238.7% (207.0–269.4%) | 300.2% (267.1–330.9%) |
| Immunity Gap ($\boldsymbol{\Delta}_{\boldsymbol{2021-2022}}\boldsymbol{)}$  1–5 years | 24.966% (24.792–25.136%) | 27.208% (27.032–27.378%) | 28.673% (28.503–28.840%) |

## **Sensitivity Analysis: Calibrated factor** $\boldsymbol{r}$ **for reduced probability of hospitalisation for post-primary RSV infections**

The calibrated value for $r$ was 0.015 (95% CrI: 0.011-0.020), meaning those with post-primary infection only have, on average, around 1.5% the probability of hospitalisation of those with primary infection in the same age-group. As shown in Table S9, one of the notable effects of this change is that the estimated FOI among older age-groups is higher than baseline. For example, the pre-pandemic (2018-2019) FOI among those 15+ years was 0.113 (95% CrI: 0.091-0.141) in the baseline model, but 1.051 (95% CrI: 0.791-1.330) in calibrated $r$. However, the FOI among those 0-4 years in 2018-2019 is estimated to be lower at 0.765 (95% CrI: 0.667-0.868) compared to 1.126 (95% CrI: 0.962-1.287) at baseline. This trend continues across the different seasons.

This means that with the calibrated $r$ model, there is a shift to more infections among the older age-groups, but many of them are inapparent due to lower probabilities of hospitalisation (due to the small value of $r$), and smaller estimated values for the probability of reporting to surveillance $\left( \rho_{j}^{Surv} \right)$ and test positive probability $\left( \rho_{j}^{V} \right)$, as shown in Figure S6.

We opted to present the baseline model due to the results of the birth cohort study in Kilifi, Kenya finding no statistically significant relationship between prior exposure and reductions in RSV disease severity when controlling for age.^7^ However, with this modification to the model, the estimated proportion of the population naïve to RSV increases from 1.7% (95% CrI: 1.6-1.9%) in 2018-2019 to 2.6% (95% CrI: 2.5-2.8%) in 2021-2022. This results in an immunity gap of 0.88% (95% CrI: 0.87-0.89%), which is consistent with the value at baseline (0.87% [95% CrI: 0.87-0.88%]).

**Table S9. Mean and 95% CrI of estimated parameters for the baseline and calibrated** $\boldsymbol{r}$ **models.** The % change in proportion naïve refers to the proportion RSV naïve in 2021-2022 versus 2018-2019.

| Parameter | Baseline | Calibrated $\boldsymbol{r}$ |
| --- | --- | --- |
| $\boldsymbol{\lambda}_{\mathbf{2018-2019}}^{\boldsymbol{A}}$ | 0.113 (0.091–0.141) | 1.051 (0.791–1.330) |
| $\boldsymbol{\lambda}_{\mathbf{2019-2020}}^{\boldsymbol{A}}$ | 0.033 (0.027–0.041) | 0.235 (0.191–0.277) |
| $\boldsymbol{\lambda}_{\mathbf{2020-2021}}^{\boldsymbol{A}}$ | 0.000 (0.000–0.000) | 0.000 (0.000–0.000) |
| $\boldsymbol{\lambda}_{\mathbf{2021-2022}}^{\boldsymbol{A}}$ | 0.080 (0.064–0.099) | 0.656 (0.513–0.798) |
| $\boldsymbol{\lambda}_{\mathbf{2018-2019}}^{\boldsymbol{C}}$ | 1.126 (0.962–1.287) | 0.765 (0.667–0.868) |
| $\boldsymbol{\lambda}_{\mathbf{2019-2020}}^{\boldsymbol{C}}$ | 0.584 (0.515–0.649) | 0.425 (0.377–0.474) |
| $\boldsymbol{\lambda}_{\mathbf{2020-2021}}^{\boldsymbol{C}}$ | 0.001 (0.001–0.001) | 0.001 (0.000–0.001) |
| $\boldsymbol{\lambda}_{\mathbf{2021-2022}}^{\boldsymbol{C}}$ | 1.406 (1.171–1.653) | 0.903 (0.780–1.036) |
| $\boldsymbol{\lambda}_{\mathbf{2018-2019}}^{\boldsymbol{Y}}$ | 0.187 (0.135–0.252) | 0.841 (0.595–1.124) |
| $\boldsymbol{\lambda}_{\mathbf{2019-2020}}^{\boldsymbol{Y}}$ | 0.025 (0.018–0.033) | 0.090 (0.069–0.111) |
| $\boldsymbol{\lambda}_{\mathbf{2020-2021}}^{\boldsymbol{Y}}$ | 0.000 (0.000–0.000) | 0.000 (0.000–0.000) |
| $\boldsymbol{\lambda}_{\mathbf{2021-2022}}^{\boldsymbol{Y}}$ | 0.095 (0.069–0.126) | 0.371 (0.277–0.469) |
| $\boldsymbol{\rho}_{\mathbf{0-6 mos.}}^{\boldsymbol{Hosp}}$ | 0.228 (0.211–0.250) | 0.289 (0.264–0.318) |
| $\boldsymbol{\rho}_{\mathbf{7-12 mos.}}^{\boldsymbol{Hosp}}$ | 0.009 (0.008–0.010) | 0.012 (0.010–0.013) |
| $\boldsymbol{\rho}_{\mathbf{1}}^{\boldsymbol{Hosp}}$ | 0.004 (0.003–0.004) | 0.006 (0.005–0.007) |
| $\boldsymbol{\rho}_{\mathbf{2}}^{\boldsymbol{Hosp}}$ | 0.002 (0.001–0.002) | 0.004 (0.003–0.004) |
| $\boldsymbol{\rho}_{\mathbf{3+}}^{\boldsymbol{Hosp}}$ | 0.000 (0.000–0.000) | 0.003 (0.002–0.004) |
| $\boldsymbol{\rho}_{\mathbf{0-4}}^{\boldsymbol{Surv}}$ | 0.221 (0.205–0.242) | 0.282 (0.259–0.308) |
| $\boldsymbol{\rho}_{\mathbf{5-14}}^{\boldsymbol{Surv}}$ | 0.202 (0.150–0.267) | 0.057 (0.046–0.072) |
| $\boldsymbol{\rho}_{\mathbf{15-64}}^{\boldsymbol{Surv}}$ | 0.171 (0.138–0.210) | 0.026 (0.022–0.031) |
| $\boldsymbol{\rho}_{\mathbf{65+}}^{\boldsymbol{Surv}}$ | 0.153 (0.123–0.187) | 0.023 (0.020–0.028) |
| $\boldsymbol{\rho}_{\mathbf{0-4}}^{\boldsymbol{V}}$ | 0.557 (0.486–0.636) | 0.701 (0.610–0.800) |
| $\boldsymbol{\rho}_{\mathbf{5-14}}^{\boldsymbol{V}}$ | 0.745 (0.521–0.962) | 0.350 (0.227–0.505) |
| $\boldsymbol{\rho}_{\mathbf{15-64}}^{\boldsymbol{V}}$ | 0.787 (0.591–0.971) | 0.162 (0.115–0.220) |
| $\boldsymbol{\rho}_{\mathbf{65+}}^{\boldsymbol{V}}$ | 0.744 (0.503–0.965) | 0.322 (0.194–0.479) |
| $\boldsymbol{\Delta}_{\mathbf{2018-2019}}^{\boldsymbol{BEFORE}}$ | 0.014 (0.013–0.016) | 0.017 (0.016–0.019) |
| $\boldsymbol{\Delta}_{\mathbf{2021-2022}}^{\boldsymbol{BEFORE}}$ | 0.023 (0.022–0.025) | 0.026 (0.025–0.028) |
| % Change Proportion Naïve | 60.8% (55.2–65.4%) | 50.9% (47.1–54.6%) |
| Immunity Gap  $\boldsymbol{(}\boldsymbol{\Delta}_{\boldsymbol{2021-2022}}\boldsymbol{)}$ | 0.874% (0.867–0.880%) | 0.880% (0.873–0.888%) |
| % Change Proportion Naïve  (1–5 years) | 161.3% (137.7–183.9%) | 108.3% (93.5–123.8%) |
| Immunity Gap ($\boldsymbol{\Delta}_{\boldsymbol{2021-2022}}\boldsymbol{)}$  1–5 years | 24.966% (24.792–25.136%) | 24.571% (24.188–24.874%) |

# **References**

1. Ministero della Salute. Hospital discharge records [Internet]. Schede di dimissione ospedaliera - SDO. Available from: https://www.salute.gov.it/portale/temi/p2_4.jsp?lingua=italiano&tema=Assistenza,%20ospedale%20e%20territorio&area=ricoveriOspedalieri

2. Istituto Superiore di Sanità. Sistema di Sorveglianza Integrata dell’Influenza [Internet]. InfluNet. [cited 2022 Aug 9]. Available from: https://w3.iss.it/site/RMI/influnet/Default.aspx

3. Italian National Institute of Statistics. Deaths : Death rate [Internet]. 2022 [cited 2022 Aug 12]. Available from: http://dati.istat.it/Index.aspx?QueryId=19671&lang=en#

4. Andeweg SP, Schepp RM, van de Kassteele J, Mollema L, Berbers GAM, van Boven M. Population-based serology reveals risk factors for RSV infection in children younger than 5 years. Sci Rep. 2021 Apr 26;11:8953.

5. Glezen WP, Taber LH, Frank AL, Kasel JA. Risk of Primary Infection and Reinfection With Respiratory Syncytial Virus. Am J Dis Child. 1986 Jun 1;140(6):543–6.

6. Kutsaya A, Teros-Jaakkola T, Kakkola L, Toivonen L, Peltola V, Waris M, et al. Prospective clinical and serological follow-up in early childhood reveals a high rate of subclinical RSV infection and a relatively high reinfection rate within the first 3 years of life. Epidemiol Infect. 2016 Jun;144(8):1622–33.

7. Ohuma EO, Okiro EA, Ochola R, Sande CJ, Cane PA, Medley GF, et al. The Natural History of Respiratory Syncytial Virus in a Birth Cohort: The Influence of Age and Previous Infection on Reinfection and Disease. Am J Epidemiol. 2012 Nov 1;176(9):794–802.

8. Paynter S, Yakob L, Simões EAF, Lucero MG, Tallo V, Nohynek H, et al. Using Mathematical Transmission Modelling to Investigate Drivers of Respiratory Syncytial Virus Seasonality in Children in the Philippines. PLOS ONE. 2014 Feb 27;9(2):e90094.

9. Public Health England. Respiratory syncytial virus (RSV): symptoms, transmission, prevention, treatment [Internet]. GOV.UK. [cited 2022 Jul 25]. Available from: https://www.gov.uk/government/publications/respiratory-syncytial-virus-rsv-symptoms-transmission-prevention-treatment/respiratory-syncytial-virus-rsv-symptoms-transmission-prevention-treatment

10. van Boven M, Teirlinck AC, Meijer A, Hooiveld M, van Dorp CH, Reeves RM, et al. Estimating Transmission Parameters for Respiratory Syncytial Virus and Predicting the Impact of Maternal and Pediatric Vaccination. J Infect Dis. 2020 Oct 7;222(Supplement_7):S688–94.

11. Gelman A, Hwang J, Vehtari A. Understanding predictive information criteria for Bayesian models. Stat Comput. 2014 Nov;24(6):997–1016.
